# A Novel Binary Hunger Games Search Algorithm with Data-Driven Repair for the Set Covering Problem

**DOI:** 10.3390/biomimetics11090645

**Published:** 2026-09-08

**Authors:** Broderick Crawford, Hugo Caballero, Gino Astorga, Felipe Cisternas-Caneo, Alan Baeza, Pablo Puga Lucero, Giovanni Giachetti, Ricardo Soto

**Affiliations:** 1 Escuela de Ingeniería Informática, Pontificia Universidad Católica de Valparaíso, Avenida Brasil 2241, Valparaíso 2362807, Chile; hugo.caballero.c@mail.pucv.cl (H.C.); alan.baeza.o@mail.pucv.cl (A.B.); pablo.puga.l@mail.pucv.cl (P.P.L.); ricardo.soto@pucv.cl (R.S.); 2 Escuela de Negocios Internacionales, Universidad de Valparaíso, Alcalde Prieto Nieto 452, Viña del Mar 2572048, Chile; gino.astorga@uv.cl; 3 Departamento de Ingeniería Industrial, Facultad de Ingeniería, Universidad del Bío-Bío, Concepción 4051381, Chile; fcisternas@ubiobio.cl; 4 Facultad de Ingeniería, Universidad Andrés Bello, Antonio Varas 880, Providencia, Santiago 7591538, Chile; giovanni.giachetti@unab.cl

**Keywords:** Set Covering Problem, Binary Hunger Games Search, feasibility repair, adaptive repair selection, combinatorial optimization, bandit-based operator selection

## Abstract

Solving problems associated with the efficient distribution and organization of resources has generated increasing interest in the scientific community. One of the most commonly used approaches consists of approximate solution techniques, which have been able to solve complex covering problems within acceptable computational time and cost. One of the benchmarks used to evaluate these approaches is the Set Covering Problem, which is an NP-hard combinatorial optimization problem. Among the techniques that have been investigated, metaheuristics play an important role. These methods are commonly developed for continuous search spaces and, in order to be applied to covering problems, must be modified to operate in discrete domains. This modification presents an important challenge: finding an appropriate transformation method that translates continuous solutions into binary solutions. This issue has been addressed through two main strategies: binarization using two-step schemes, and, in our proposal, the use of repair operators orchestrated according to their performance through an Adaptive Repair Selection Mechanism based on the multi-armed bandit framework. To evaluate our proposal, we selected the Binary Hunger Games Search metaheuristic because the relative quality of each individual determines its hunger level, which in turn regulates the movement of the population and the influence of the best solution found. Infeasible solutions are handled through a set of Tabu Search-based repair operators. Instead of applying a single repair rule throughout the entire execution, the proposed approach dynamically selects among these operators according to their observed contribution during the search. Each repair operator also incorporates Tabu memory to discourage repetitive decisions during feasibility restoration. The experiments were conducted using the classical Beasley benchmark instances for the Set Covering Problem.

## 1. Introduction

Resource management problems and the optimization of resource allocation arise in various areas of human activity; they have historically appeared in projects of different natures that deal with resource administration, such as logistics, telecommunications, road infrastructure, and the health sector, among others. The challenge we face when efficiently allocating these assets lies in the combinatorial complexity inherent in their use; that is, how to find high-quality solutions in search spaces that grow exponentially with the size of the problem, without incurring prohibitive computation times. Exact methods allow for obtaining optimal solutions and certifying their optimality. Nevertheless, for certain problem instances, the growth of the search space and the cost of closing the optimality gap may lead to high time and memory requirements. When these requirements exceed the available computational budget, metaheuristics (MH) constitute an alternative for obtaining approximate solutions, although they do not guarantee reaching the global optimum [1,2].

Among the most widely used MH are population-based methods inspired by natural phenomena, which employ a set of candidate solutions and update mechanisms derived from biological, social, or physical models [3,4]. For example, Ant Colony Optimization uses computational mechanisms inspired by the indirect communication observed in ant colonies and has been applied to combinatorial optimization problems [5,6]. However, natural inspiration does not by itself determine the effectiveness of a method; its behavior depends on the operators implemented and their interaction with the characteristics of the problem under consideration.

This work addresses the Set Covering Problem (SCP) through a binary adaptation of Hunger Games Search (HGS) that incorporates adaptive repair-operator selection. HGS is a population-based MH originally formulated for continuous search spaces. Because its search mechanism is inspired by hunger-driven interactions among animals, HGS can be situated within the broader family of nature-inspired, or biomimetic, optimization methods [4,7]. Its update rules use variables associated with the hunger level of individuals and information from the best solutions found by the population [7]. In the original study, HGS was evaluated using continuous benchmark functions and engineering design problems. Its selection as the continuous search engine in this study is motivated by its fitness-dependent adaptive dynamics, in which hunger levels and adaptive weights regulate the influence of the best solution and the balance between exploration and exploitation. These characteristics provide a suitable search framework for studying how binarization and feasibility restoration affect the behavior of a continuous metaheuristic when transferred to a constrained binary problem. Its application to the SCP, however, requires additional mechanisms because the problem involves binary decision variables and coverage constraints. The proposed adaptation therefore comprises two components: a binarization scheme and a procedure for repairing infeasible solutions. The binarization scheme maps the continuous positions generated by HGS to binary vectors [8,9]. Because this transformation does not guarantee satisfaction of the coverage constraints, infeasible binary solutions are processed using repair operators.

A question then arises: which repair operator should be used? The operators described in the literature apply different criteria to restore feasibility and eliminate redundancy. However, in the reviewed literature, we did not identify a widely accepted classification for the SCP. For this reason, in this work, we organize them according to the way in which they reestablish coverage and remove redundant columns [10,11,12]. Building on this basis, we propose complementing the repair process with a local search that incorporates short-term Tabu memory [13,14]. The procedure removes selected columns and restores coverage through a greedy rule based on the relationship between cost and coverage contribution [10]. This proposal is supported by studies that have incorporated Tabu strategies into search methods for the SCP, although not specifically as repair operators [15,16].

HGS belongs to the family of biomimetic optimization algorithms and is inspired by the hunger-driven behavior of animals. In this model, individuals modify their positions according to hunger levels, search pressure, and the influence of the best solutions found during the optimization process. From a biomimetic perspective, the hunger mechanism provides an adaptive metaphor for regulating the balance between exploration and exploitation, since candidate solutions are guided by both individual search dynamics and collective information obtained from the population. This mechanism allows the algorithm to alternate between exploration and exploitation through adaptive movement rules, making it suitable for complex search spaces [7].

To address this issue, the repair phase is treated as an adaptive component of the search process. A Multi-Armed Bandit (MAB) mechanism [17] is employed to dynamically select the most appropriate repair operator from a predefined set according to its observed contribution to solution quality. This strategy follows the principles of adaptive operator selection and allows the algorithm to avoid relying on a single fixed repair rule. In this way, the repair strategy becomes part of the search behavior itself, influencing not only the restoration of feasibility but also the robustness, stability, and convergence of the binary metaheuristic.

The remainder of this paper is organized as follows. Section 2 introduces the SCP, its mathematical formulation, and an illustrative application example. Section 3 presents the original HGS algorithm, including its hunger mechanism, adaptive weights, and exploration and exploitation phases. Section 4 describes the binary adaptation process, including the V3-ELIT binarization scheme, the feasibility repair phase, and the adaptive repair selection strategy. Section 5 details the proposed Binary HGS with Adaptive Repair Selection. Section 6 presents the experimental setup, solution quality analysis, convergence behavior, and statistical comparison between the evaluated repair strategies. Section 7 summarizes the novelty and main contributions of the proposed approach. Finally, Section 8 presents the main conclusions, and Section 9 discusses future research directions.

## 2. Set Covering Problem

The SCP was chosen because it exposes the main difficulty considered in this work: after transforming a continuous solution into a binary vector, the resulting solution is not necessarily feasible. Therefore, the problem allows us to evaluate not only the performance of the binary version of HGS but also the role of the repair operators in restoring coverage constraints. We use the classical Beasley instances because they are widely used in SCP studies and provide a common reference for comparing results.

### 2.1. Mathematical Formulation of the Set Covering Problem

Let M={1,2,…,m} be the set of elements that must be covered, and let N={1,2,…,n} be the set of available subsets. Each subset j∈N has an associated cost $c_{j}$, and the binary parameter $a_{ij}$ indicates whether element i∈M is covered by subset j∈N. Thus, $a_{ij}=1$ if subset *j* covers element *i*, and $a_{ij}=0$ otherwise.

The decision variable $x_{j}$ is defined as follows:

(1) $$x_{j}=\left\{\begin{array}{cc} 1, & \mathrm{if}\;\mathrm{subset}\;j\;\mathrm{is}\;\mathrm{selected},\\ 0, & \mathrm{otherwise}. \end{array}\right.$$

The classical Set Covering Problem can be formulated as follows:

(2) $$\min\sum_{j=1}^{n}c_{j}x_{j}$$

subject to:

(3) $$\sum_{j=1}^{n}a_{ij}x_{j}\geq 1,\;\forall i\in M$$

(4) $$x_{j}\in \left\{0,1\right\},\;\forall j\in N$$

The objective function in Equation (2) minimizes the total cost of the selected subsets. The coverage constraints in Equation (3) ensure that each element *i* is covered by at least one selected subset. Finally, Equation (4) defines the binary nature of the decision variables.

### 2.2. Illustrative Example with Seven Demand Zones

Consider a hospital location planning problem with seven demand zones, M={1,2,3,4,5,6,7}, and five candidate hospital locations, N={1,2,3,4,5}. Each candidate hospital can cover a subset of demand zones according to a maximum travel distance or service time criterion. The coverage relationships and associated costs for the five candidate hospital locations are summarized in Table 1.

In this example, a value of 1 indicates that the candidate hospital can cover the corresponding demand zone, while a value of 0 indicates that it cannot. For instance, candidate hospital $H_{1}$ covers zones 1, 2, and 4, whereas candidate hospital $H_{4}$ covers zones 3, 6, and 7.

The objective is to select a subset of candidate hospitals that covers all seven demand zones while minimizing the total cost. The mathematical model is:

$$\min120x_{1}+100x_{2}+150x_{3}+90x_{4}+110x_{5}$$

subject to:

$$x_{1}+x_{3}\geq 1\;(\mathrm{Zone}\;1)$$

$$x_{1}+x_{2}+x_{5}\geq 1\;(\mathrm{Zone}\;2)$$

$$x_{2}+x_{4}\geq 1\;(\mathrm{Zone}\;3)$$

$$x_{1}+x_{3}\geq 1\;(\mathrm{Zone}\;4)$$

$$x_{2}+x_{3}+x_{5}\geq 1\;(\mathrm{Zone}\;5)$$

$$x_{3}+x_{4}\geq 1\;(\mathrm{Zone}\;6)$$

$$x_{4}+x_{5}\geq 1\;(\mathrm{Zone}\;7)$$

$$x_{j}\in \left\{0,1\right\},\;j=1,\ldots ,5.$$

A feasible solution is, for example, selecting hospitals $H_{1}$, $H_{2}$, and $H_{4}$:

$$x_{1}=1,\;x_{2}=1,\;x_{3}=0,\;x_{4}=1,\;x_{5}=0.$$

This solution covers all seven demand zones with a total cost of:

120+100+90=310.

This illustrative example shows the basic structure of the Set Covering Problem in a simple hospital location planning scenario. However, in real-world applications, the number of demand zones and candidate locations can grow significantly, leading to large-scale instances with many binary decisions and coverage constraints [18]. In such cases, obtaining an exact optimal solution may require high computational effort, especially when decisions must be made within limited planning times. Therefore, having at least one high-quality approximate solution becomes highly valuable, since it can provide decision makers with a feasible and cost-effective coverage plan. This is one of the main reasons why metaheuristic approaches are relevant for the SCP: they can explore large solution spaces and generate competitive feasible solutions when exact methods become impractical.

## 3. Hunger Games Search

Hunger Games Search (HGS) is a population-based metaheuristic proposed by Yang et al. [7], inspired by hunger-driven foraging behavior. In HGS, each individual represents a candidate solution in a continuous search space. During the optimization process, individuals update their positions according to their fitness values, hunger levels, adaptive weights, and the best solutions found by the population.

HGS can be described by the following three components: the hunger mechanism with its adaptive weights, the exploration phase, and the exploitation phase.

### 3.1. Hunger Mechanism and Adaptive Weights

The Hunger Games Search algorithm incorporates a hunger mechanism to regulate the movement of each individual according to its relative fitness within the population. Let F(i) be the fitness value of individual *i*, BF the best fitness value, and WF the worst fitness value in the current population. These values are defined as:

(5) $$BF=\min_{i=1,\ldots ,N}F\left(i\right),$$

(6) $$WF=\max_{i=1,\ldots ,N}F\left(i\right).$$

For each individual, the temporary hunger value TH is computed as:

(7) $$TH=\frac{F(i)-BF}{WF-BF}\cdot r_{6}\cdot 2\cdot \left(UB-LB\right),$$

where $r_{6}$ is a random number uniformly distributed in [0,1], and UB and LB are the upper and lower bounds of the search space, respectively.

The hunger increment *H* is defined as:

(8) $$H=\left\{\begin{array}{cc} LH\cdot (1+r), & TH<LH,\\ TH, & TH\geq LH, \end{array}\right.$$

where LH is a lower bound for the hunger value and *r* is a random number in [0,1].

The hunger value of each individual is then updated as follows:

(9) $$hungry\left(i\right)=\left\{\begin{array}{cc} 0, & F(i)=BF,\\ hungry(i)+H, & F(i)\neq BF. \end{array}\right.$$

The total hunger of the population is given by:

(10) $$SHungry=\sum_{i=1}^{N}hungry\left(i\right).$$

Based on the hunger value, HGS defines two adaptive weights. The first weight, $W_{1,i}$, controls the influence of the best individual:

(11) $$W_{1,i}=\left\{\begin{array}{cc} \frac{hungry(i)\cdot N}{SHungry}\cdot r_{4}, & r_{3}<l,\\ 1, & r_{3}\geq l, \end{array}\right.$$

where $r_{3}$ and $r_{4}$ are random numbers in [0,1]; *N* is the population size, and *l* is a control parameter.

The second weight, $W_{2,i}$, regulates the movement amplitude around the best solution:

(12) $$W_{2,i}=\left(1-\exp\left(-|hungry(i)-SHungry|\right)\right)\cdot r_{5}\cdot 2,$$

where $r_{5}$ is a random number in [0,1]. These adaptive weights allow HGS to modify the search behavior of each individual according to its hunger state and relative fitness.

### 3.2. Exploration Phase in HGS

The exploration phase in HGS is associated with the first movement rule of the algorithm. In this case, the individual updates its position based on its current location without using the best solution found by the population. This movement introduces random perturbations and promotes diversification in the search space.

Let $X_{i}\left(t\right)$ be the position of individual *i* at iteration *t*. The exploratory movement is defined as:

(13) $$X_{i}\left(t+1\right)=X_{i}\left(t\right)\cdot \left(1+\mathrm{randn}\left(1\right)\right),$$

where randn(1) is a random value sampled from a normal distribution. This rule is applied when:

(14) $$r_{1}<l,$$

where $r_{1}$ is a random number in [0,1] and *l* is a control parameter. The parameter *l* regulates the probability of applying the exploratory movement, and is updated as:

(15) $$l=l_{\max}-\left(l_{\max}-l_{\min}\right)\frac{t}{T},$$

where $l_{\max}$ and $l_{\min}$ are the maximum and minimum values of *l*, *t* is the current iteration, and *T* is the maximum number of iterations.

As the search progresses, the value of *l* decreases, reducing the frequency of random exploratory movements and allowing the algorithm to gradually shift toward exploitation.

### 3.3. Exploitation Phase in HGS

The exploitation phase in HGS is guided by the best solution found in the current population. This phase corresponds to the second and third movement rules of the algorithm, where each individual moves around the best individual using hunger-based adaptive weights.

Let $X_{b}\left(t\right)$ be the best individual at iteration *t*, and let $X_{i}\left(t\right)$ be the current position of individual *i*. The complete position update rule of HGS is given by:

(16) $$X_{i}\left(t+1\right)=\left\{\begin{array}{cc} X_{i}\left(t\right)\cdot \left(1+\mathrm{randn}\left(1\right)\right), & r_{1}<l,\\ W_{1,i}\cdot X_{b}\left(t\right)+R\cdot W_{2,i}\cdot \left|X_{b}\left(t\right)-X_{i}\left(t\right)\right|, & r_{1}>l,\;r_{2}>E_{i},\\ W_{1,i}\cdot X_{b}\left(t\right)-R\cdot W_{2,i}\cdot \left|X_{b}\left(t\right)-X_{i}\left(t\right)\right|, & r_{1}>l,\;r_{2}<E_{i}. \end{array}\right.$$

The exploitation movements are therefore defined as:

(17) $$X_{i}\left(t+1\right)=W_{1,i}\cdot X_{b}\left(t\right)+R\cdot W_{2,i}\cdot \left|X_{b}\left(t\right)-X_{i}\left(t\right)\right|,$$

(18) $$X_{i}\left(t+1\right)=W_{1,i}\cdot X_{b}\left(t\right)-R\cdot W_{2,i}\cdot \left|X_{b}\left(t\right)-X_{i}\left(t\right)\right|.$$

The parameter $E_{i}$ controls the selection between the two exploitation movements and is defined as follows:

(19) $$E_{i}=\mathrm{sech}\left(|F(i)-BF|\right),$$

where sech(·) is the hyperbolic secant function.

The stochastic movement factor *R* is computed as:

(20) R=2·shrink·rand−shrink,

where rand is a random number in [0,1]. The shrink factor is defined as:

(21) $$shrink=2\left(1-\frac{t}{T}\right),$$

where *t* is the current iteration and *T* is the maximum number of iterations. Since shrink decreases as the algorithm progresses, the movement range is gradually reduced. This allows HGS to perform broader movements during the early iterations and more focused movements near promising regions during the later stages.

### 3.4. Algorithmic Structure of Hunger Games Search

Table 2 summarizes the main stages of the original Hunger Games Search algorithm, indicating the role of each stage in the initialization, hunger update, adaptive weighting, position update, and final selection of the best solution.

The procedure described in Algorithm 1 represents the core of the original HGS metaheuristic. The algorithm is organized into sequential stages that start with the initialization of the population and hunger values, followed by the evaluation of individuals through the objective function of the problem being solved. Based on these fitness values, HGS updates the best and worst individuals, computes the hunger levels, derives the adaptive weights, and then applies the position update rules. This last stage explicitly defines the exploration and exploitation behaviors of the method, allowing the population to alternate between random diversification and guided movements around the best solution found so far.
**Algorithm 1** Pseudo-code of the Original Hunger Games Search (HGS)**Require:** *N*, *T*, *l*, *D*, SHungry **Ensure:** BF, $X_{b}$    **Stage 1: Initialization**   1:Initialize the positions of individuals $X_{i}$, i=1,2,…,N  2:Initialize the hunger values hungry(i)  3:**while** t≤T **do****Stage 2: Fitness Evaluation**  4:   Calculate the fitness of all individuals  5:   Update BF, WF, $X_{b}$, and BI using Equations (5) and (6)**Stage 3: Hunger Update**  6:   Calculate hungry(i) using Equation (9)**Stage 4: Adaptive Weight Update**  7:   Calculate $W_{1}$ using Equation (11)  8:   Calculate $W_{2}$ using Equation (12)**Stage 5: Position Update**  9:   **for** each individual **do**10:      Calculate *E* using Equation (19)11:      Update *R* using Equation (20)12:      **if** $r_{1}<l$ **then**   **Exploration phase**13:         Update position using Equation (13)14:      **else**   **Exploitation phase**15:         Update position using Equation (17) or Equation (18)16:      **end if**17:   **end for**18:   t←t+119:**end while**20:**return** BF, $X_{b}$

## 4. Binary Adaptation via Binarization and Adaptive Repair

The original HGS algorithm operates in a continuous search space. However, the Set Covering Problem requires binary decision variables and feasible solutions that satisfy all coverage constraints. Therefore, a direct application of HGS to the SCP is not possible without an adaptation mechanism. Following the binary adaptation framework proposed by Crawford et al. [19], we apply the same methodological criteria, considering both the binarization strategy and the adaptive selection of feasibility-repair operators. Consequently, the adaptation of HGS is built upon two essential components: binarization and adaptive feasibility repair.

### 4.1. Two-Step Binarization Scheme

**Figure 1:**
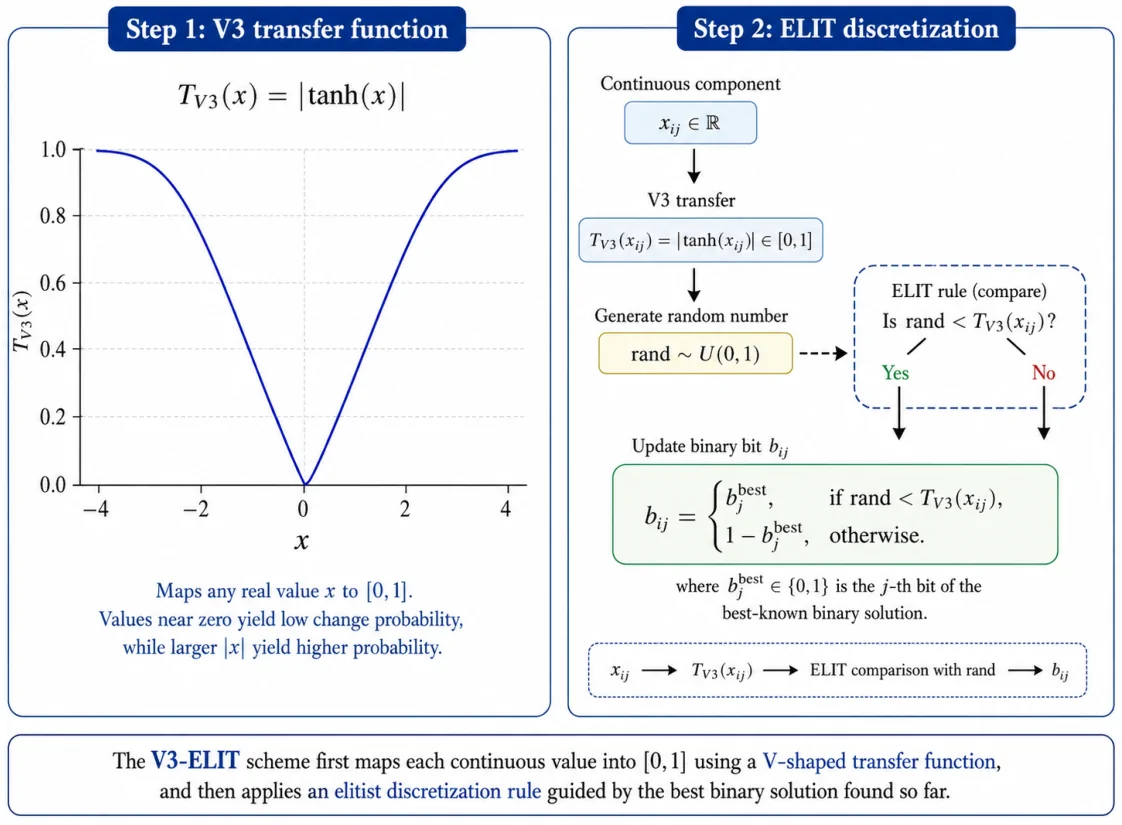
Two-step V3-ELIT binarization scheme. The V3 transfer function maps each continuous value into the interval [0,1], and the ELIT discretization rule uses the corresponding bit of the best binary solution found so far to define the final binary decision.

Figure 1 illustrates the two-step binarization scheme used to transform the continuous values generated by HGS into binary decisions. In the first step, each continuous component $x_{ij}$ is mapped into the interval [0,1] using the V3 transfer function, $T_{V3}\left(x\right)=\left|\tanh\left(x\right)\right|$. This value represents the probability of following the elitist discretization criterion. In the second step, the ELIT rule compares $T_{V3}\left(x_{ij}\right)$ with a random value uniformly generated in [0,1]. If the condition is satisfied, the binary component adopts the corresponding bit of the best solution found so far; otherwise, it takes the complementary value. Therefore, the binarization process is guided by the best known binary solution while still preserving stochastic variation during the search.

### 4.2. Feasibility Repair

Repair is the process in which each binary vector solution is evaluated to determine whether it satisfies the problem’s coverage constraints. Its role is to transform the proposed solution and infeasible binary vector into a feasible solution before evaluating it against the objective function. This process is often referred to as solution restoration and has a considerable impact since the selected and aggregated subsets modify the structure of the final solution [20,21] and the value of the solution. Given the critical nature and frequency of this process, we have focused on evaluating the impact of various Repair Operators (ORE), which can produce different feasible solutions from the same infeasible vector. Each ORE has a distinct architecture, and instead of using a single fixed repair rule, the proposed approach selects among multiple repair operators based on their observed performance during the search. This strategy follows the data-driven repair mechanism introduced by Crawford et al. [19], where adaptive repair selection yielded robust results for the SCP.

### 4.3. Repair Operators Used

Tabu Search-based operators were selected because the SCP repair process can fall into repetitive column-selection patterns. When repairing infeasible solutions, criteria based on cost and coverage may repeatedly favor similar columns, producing similar or redundant feasible solutions. To reduce this behavior, short-term Tabu memory is incorporated into the repair process to discourage recently explored removal decisions and promote alternative feasible restorations [13,14]. In this work, Tabu Search is used as a memory mechanism within the repair procedure rather than as an independent optimization algorithm. A comprehensive treatment of the method was provided by Glover and Laguna [22].

Let $c_{j}$ denote the cost of column *j*, and let

(22) $$q_{j}=\sum_{i\in U}a_{ij},$$

where $q_{j}$ is the number of currently uncovered rows that can be covered by column *j*, *U* is the set of uncovered rows, $a_{ij}=1$ if column *j* covers row *i*, and $a_{ij}=0$ otherwise. Based on this definition, the three Tabu-based repair operators are formulated as follows:

1. Tabu Complex Stochastic (TCS).
  The tabu_complex_stochastic operator evaluates each candidate column using the cost–coverage ratio
  (23) $$\rho_{j}=\frac{c_{j}}{q_{j}},$$
  where $\rho_{j}$ represents the cost–coverage ratio of column *j*, $c_{j}$ is its original cost, and $q_{j}$ is the number of currently uncovered rows that the column can cover. A smaller value of $\rho_{j}$ therefore represents a more favorable relationship between cost and additional coverage.
  The candidate columns are ranked according to Equation (23). The five columns with the lowest values of $\rho_{j}$, or all candidates when fewer than five are available, form a restricted candidate set. One column is then selected uniformly at random from this set and incorporated into the solution. The procedure is repeated until all rows are covered.
2. Tabu Complex Penalized (TCP).
  The tabu_complex_penalized operator incorporates a frequency penalty into the cost–coverage criterion:
  (24) $$\rho_{j}^{P}=\frac{c_{j}\left(1+\alpha p_{j}\right)}{q_{j}},\;\alpha =0.2,$$
  where $\rho_{j}^{P}$ is the penalized cost–coverage ratio of column *j*, $c_{j}$ is its original cost, $p_{j}$ is the accumulated number of times that column *j* has been selected by this repair mechanism, α controls the strength of the frequency penalty, and $q_{j}$ represents the contribution of column *j* to the currently uncovered rows.
  The term $\left(1+\alpha p_{j}\right)$ progressively increases the effective cost of frequently selected columns. The candidate with the smallest value of $\rho_{j}^{P}$ is selected and incorporated into the solution, and its frequency counter $p_{j}$ is increased by one. The procedure continues until feasibility is restored.
3. Tabu Complex Stochastic Drop (TCS-D).
  The tabu_complex_stochastic_drop operator first restores feasibility using the stochastic cost–coverage mechanism defined in Equation (23). Once a feasible solution has been obtained, selected columns are examined in decreasing order of cost to identify redundant columns.
  A selected column *j* can be removed when
  (25) $$\sum_{k\neq j}a_{ik}x_{k}\geq 1,\;\forall i\;\mathrm{such}\;\mathrm{that}\;a_{ij}=1,$$
  where *j* is the column being evaluated for removal, *i* denotes a row covered by column *j*, *k* indexes all remaining columns, $a_{ik}$ indicates whether column *k* covers row *i*, and $x_{k}=1$ indicates that column *k* is currently selected.
  Therefore, $\sum_{k\neq j}a_{ik}x_{k}$ represents the coverage of row *i* provided by the remaining selected columns after excluding column *j*. If this value is at least one for every row covered by *j*, the column is redundant and can be removed without violating feasibility. Since columns are evaluated from highest to lowest cost, the procedure gives priority to eliminating expensive redundant columns.

After the corresponding feasibility-restoration procedure, the three operators use the same Tabu Search refinement. The implementation uses a Tabu tenure of 5, a maximum of 15 Tabu iterations, a neighborhood sample of up to 10 active columns, and at most 3 column additions during local feasibility restoration. At each Tabu iteration, sampled active columns are temporarily removed to generate neighboring solutions. If coverage is lost, feasibility is locally restored using the minimum cost–coverage ratio $c_{j}/q_{j}$. Among the feasible neighbors generated, the solution with the lowest objective value is selected, while recently removed columns remain Tabu according to the defined tenure.

### 4.4. Adaptive Repair Selection Strategy

The Adaptive Repair Selection Mechanism (ARSM) is based on the multi-armed bandit framework, whose theoretical foundations can be traced back to Robbins [23], with early probabilistic antecedents related to Thompson [24]. In this framework, each available alternative is treated as an arm, and its utility is progressively estimated from the rewards obtained after its selection. Following the principles of adaptive operator selection, the mechanism determines which feasibility repair operator should be applied when an infeasible binary solution is generated during the BHGS search process. In this study, the available arms correspond to the three Tabu-based repair operators defined in Table 3. The estimated utility of the selected operator is updated according to the improvement obtained. In this way, the algorithm does not rely on a fixed repair rule; instead, it learns during execution which repair operator contributes more effectively to improving the best feasible solution.

Let $R=\{R_{1},R_{2},\ldots ,R_{K}\}$ be the set of available repair operators. In this study, R is composed of three Tabu-based repair operators: Tabu complex stochastic, Tabu complex penalized, and Tabu complex stochastic drop [13]. At iteration *t*, when an infeasible binary solution is generated, one repair operator $a_{t}\in R$ is selected according to an ϵ-greedy policy:

(26) $$a_{t}=\left\{\begin{array}{cc} \mathrm{random}\;\mathrm{repair}\;\mathrm{operator}\;\mathrm{from}\;R, & \mathrm{if}\;u<\epsilon ,\\ \arg\max_{a\in R}Q\left(a\right), & \mathrm{otherwise}, \end{array}\right.$$

where u∼U(0,1), ϵ controls the exploration rate, and Q(a) represents the estimated utility of repair operator *a*. The first case promotes exploration by testing different repair operators, while the second case exploits the operator with the highest accumulated evidence of performance.

After applying the selected repair operator, the reward is computed from the improvement in the best objective value obtained by the algorithm. Let $f_{best}^{\left(t-1\right)}$ and $f_{best}^{\left(t\right)}$ be the best objective values before and after applying the selected repair operator at iteration *t*. The reward is defined as:

(27) $$r_{t}=f_{best}^{\left(t-1\right)}-f_{best}^{\left(t\right)}.$$

For the SCP minimization problem, $r_{t}>0$ indicates that the application of the selected repair operator contributed to improving the global best solution. If the operator produces a feasible solution but does not improve the global best, then $f_{best}^{\left(t\right)}=f_{best}^{\left(t-1\right)}$ and, consequently, $r_{t}=0$. In this case, the operator is still considered to have been selected, and its selection count is increased, but it receives no positive reward. The zero reward is nevertheless incorporated into the utility update, allowing the estimated performance of the operator to reflect both improving and non-improving applications.

The operator’s utility is updated using an incremental sample-average rule:

(28) $$Q\left(a_{t}\right)\leftarrow Q\left(a_{t}\right)+\frac{r_{t}-Q\left(a_{t}\right)}{N(a_{t})},$$

where $N(a_{t})$ is the number of times operator $a_{t}$ has been selected. This update favors operators that repeatedly improve the global best solution, while non-improving applications contribute a zero reward to their estimated utility. The ϵ-greedy policy in Algorithm 2 maintains the balance between exploration and exploitation.
**Algorithm 2** Multi-Armed Bandit Repair Selection and Reward Update**Require:** Repair operator set $R=\{R_{1},R_{2},\ldots ,R_{K}\}$, exploration rate ϵ, estimated utilities $Q(a_{i})$, selection counts $N(a_{i})$, and current best objective value $f_{\mathrm{best}}^{\left(t-1\right)}$ **Ensure:** Selected repair operator $a_{t}$ and updated utility $Q(a_{t})$
  1:Draw u∼U(0,1)  2:Select the repair operator $a_{t}$ according to Equation (26)  3:Apply the selected repair operator $a_{t}$  4:Obtain the new best objective value $f_{\mathrm{best}}^{\left(t\right)}$  5:Compute the reward $r_{t}$ according to Equation (27)  6:Update the selection count $N\left(a_{t}\right)\leftarrow N\left(a_{t}\right)+1$  7:Update the estimated utility $Q(a_{t})$ according to Equation (28)  8:**return** $a_{t}$, $Q(a_{t})$, $N(a_{t})$


**Figure 2:**
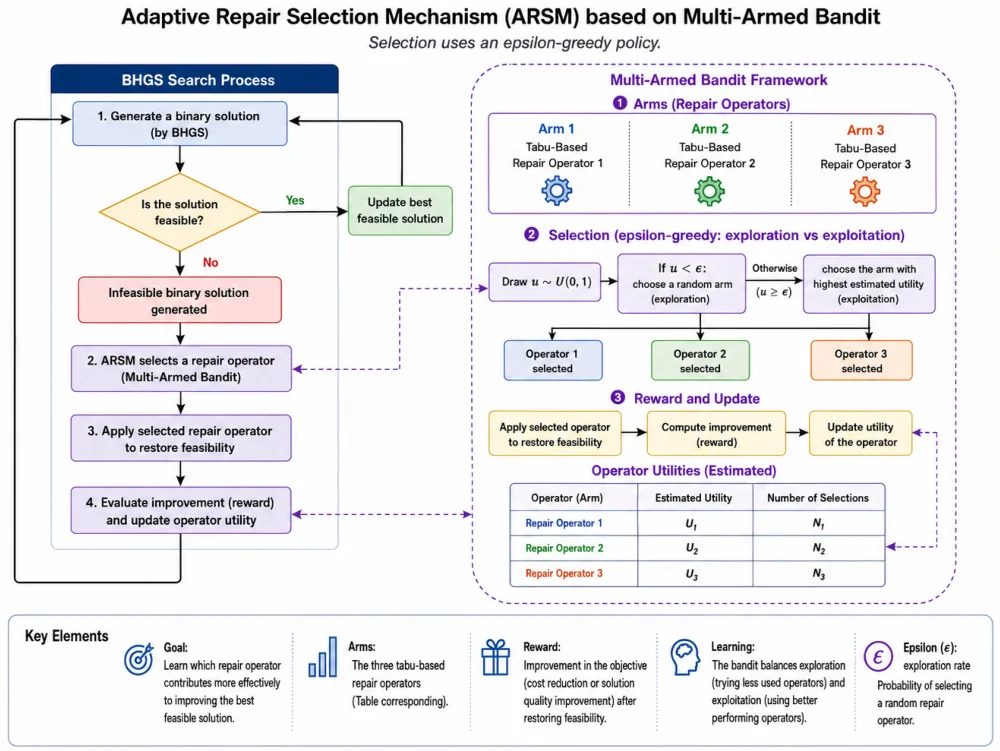
Integration of the different components of the BHGS solution, providing a comprehensive view of its structure and operation.

Figure 2 presents the integration of the different components of the BHGS solution, allowing its structure and operation to be visualized comprehensively.

## 5. Binary HGS

Binary Hunger Games Search (BHGS) extends the original continuous space HGS metaheuristic described in Algorithm 1 to the binary domain required to solve Set Covering Problems. HGS is responsible for generating and updating candidate solutions in the continuous search space. After each movement, the resulting continuous vector is transformed into a binary solution using the V3-ELIT binarization scheme. Then, the binary solution is verified against the SCP coverage constraints. If the solution is infeasible, a repair operator is selected through ARMS and applied to restore feasibility. Finally, the contribution of the repair operator is used to update its reward, allowing the mechanism to progressively adapt operator selection during the search.

Table 4 summarizes the notation used to describe the proposed Binary HGS with Adaptive Repair Selection algorithm. The variables include the indices used to identify individuals, decision variables, repair operators, and iterations, as well as the main elements involved in the continuous HGS search, the V3-ELIT binarization process, the feasibility repair stage, and the reward-based adaptive selection mechanism.

## 6. Experimental Results

This section presents the experimental results obtained by the Binary Hunger Games Search with Adaptive Repair Selection algorithm for the Set Covering Problem. The experimental results were analyzed in terms of solution quality, robustness, and convergence behavior on the considered benchmark instances.

### 6.1. Methodology

The experimental study was conducted using the solver described in Algorithm 3 and a set of classical SCP benchmark instances from the OR-Library, originally introduced by Beasley [20]. The characteristics of these instances are summarized in Table 5 and include the sets 4, 5, 6, A, B, C, D, NRE, NRF, NRG, and NRH, which cover a wide range of problem sizes, coverage densities, and cost structures. Beyond reporting the best solutions obtained, the study includes a comparative analysis based on the median solution quality and the convergence iteration (CI). This comparison is relevant because it allows the performance of the complex and Tabu Search-based repair operators to be evaluated in terms of both robustness and convergence behavior, rather than only by their best-case results.
**Algorithm 3** Binary Hunger Games Search with Adaptive Repair Selection**Require:** SCP instance, population size *N*, maximum iterations *T*, dimension *D*, V3-ELIT binarization scheme, repair operator set R **Ensure:** Best feasible binary solution $B^{best}$ and best fitness value BF
  1:Initialize the continuous HGS population $X_{i}$, i=1,…,N  2:Initialize hunger values and HGS control parameters  3:Initialize the estimated utility $Q(R_{k})$ and selection count $N(R_{k})$ for each repair operator $R_{k}\in R$  4:**for** t=1 to *T* **do**  5:   Update the continuous population using the original HGS procedure in Algorithm 1  6:   **for** each individual $X_{i}$ **do**  7:      Apply the V3-ELIT binarization scheme to obtain binary solution $B_{i}$  8:      Test the feasibility of $B_{i}$ according to the SCP coverage constraints  9:      **if** $B_{i}$ is infeasible **then**10:         Select a repair operator $R_{t}$ using Algorithm 211:         Apply $R_{t}$ to obtain a feasible solution ${\hat{B}}_{i}$12:      **else**13:         ${\hat{B}}_{i}\leftarrow B_{i}$14:      **end if**15:      Evaluate ${\hat{B}}_{i}$ using the SCP objective function16:      **if** ${\hat{B}}_{i}$ improves the current best solution **then**17:         Update $B^{best}$ and BF18:      **end if**19:      **if** a repair operator was applied **then**20:         Compute the reward obtained by $R_{t}$21:         Update $Q(R_{t})$ and $N(R_{t})$ using Algorithm 222:      **end if**23:   **end for**24:**end for**25:**return** $B^{best}$, BF


For each benchmark and experimental configuration, 31 independent runs were performed. In all experiments, the same population size, maximum number of BHGS iterations, benchmark instances, and stopping criterion were used for both repair configurations. These conditions ensure an equivalent outer search budget. However, the internal computational effort of the repair procedures is not identical. The Tabu Search-based repair operators perform an additional neighborhood exploration after feasibility restoration, whereas the complex repair operators do not include this internal search stage. Consequently, the comparison controls the main BHGS experimental conditions but does not assume equivalent execution time or identical internal computational cost.

The complex repair operators were first incorporated as a methodological baseline since they were previously used under the same data-driven repair principle in the DOA-based approach reported by Crawford et al. [19]. Their inclusion allows the proposed Tabu Search-based repair operators to be evaluated under a controlled comparison within the same BHGS framework. Therefore, both repair strategies were assessed using the same benchmark instances, stopping criterion, adaptive selection scheme, population size, and maximum number of BHGS iterations.

For the experimental evaluation of the proposed metaheuristic, we used a set of performance indicators commonly reported in recent SCP studies. These indicators allow the quality, stability, and robustness of the solutions obtained across independent runs to be assessed objectively. The following measures were considered:

- Relative Percentage Deviation (RPD), which measures the percentage deviation of the solution cost obtained by the algorithm with respect to the known optimal cost of the benchmark instance. It is computed as follows:
  (29) $$RPD=\frac{C_{alg}-C_{opt}}{C_{opt}}\times 100$$
  where $C_{alg}$ is the solution cost obtained by the algorithm, and $C_{opt}$ is the known optimal cost of the benchmark instance.
- Coefficient of Variation (CV), which evaluates the stability of the algorithm across independent runs by measuring the ratio between the standard deviation and the mean solution cost. A lower CV indicates greater stability across independent runs. It is computed as follows:
  (30) $$CV=\frac{\sigma }{\mu }\times 100$$
  where σ is the standard deviation of the solution costs, and μ is the mean solution cost across independent runs.
- Median solution cost, which is reported as an indicator of the typical performance of the algorithm across independent runs since it is less sensitive to extreme values than the mean. It is computed as follows:
  (31) $$C_{median}=\mathrm{median}\left(C_{1},C_{2},\ldots ,C_{R}\right)$$
  where $C_{r}$ is the solution cost obtained in run *r*, and *R* is the total number of independent runs.
- The convergence iteration (CI) was used to measure how early the algorithm reaches its final best solution. It is defined as:
  (32) $$CI=t_{\mathrm{best}}$$
  where $t_{\mathrm{best}}$ is the first iteration in which the final best fitness value is reached. Therefore, lower CI values indicate faster convergence.
- To evaluate how representative the best solution is with respect to the central behavior of the algorithm, the difference between the median-based RPD and the best-based RPD was computed as follows:
  (33) $$\Delta RPD=RPD_{median}-RPD_{best}$$
  where $RPD_{median}$ represents the relative percentage deviation computed from the median solution cost, and $RPD_{best}$ represents the relative percentage deviation computed from the best solution cost.
  For interpretation purposes in this study, the values of ΔRPD were grouped into four categories, as shown in Table 6. These categories are not intended to define universal thresholds but to support the analysis of how representative the best solution is with respect to the median behavior observed across the 31 independent runs.

### 6.2. Parameter Setting

In order to determine the value of the exploration parameter ϵ used in ARSM, preliminary experiments were conducted on a representative subset of 18 SCP instances under the same experimental conditions. As shown in Table 7, ϵ=0.10, ϵ=0.15, and ϵ=0.20 achieved the same average $RPD_{\mathrm{best}}$ of 0.088% and reached the best-known solution in 17 of the 18 instances. Therefore, the selection of ϵ=0.15 was not based on the best-solution indicator alone. This value achieved the lowest average $RPD_{\mathrm{median}}$ (0.215%), the lowest average coefficient of variation (0.264%), and the highest number of instances reaching the optimum in terms of median performance (15/18). These results indicate a more consistent typical behavior across independent runs. Although ϵ=0.15 required a higher average execution time than ϵ=0.10 and ϵ=0.20, it was selected because it provided the strongest combination of median solution quality and stability among the evaluated configurations.

The parameter configuration used in the proposed approach is summarized in Table 8. The table includes the main parameters of the metaheuristic, the binarization scheme, and the configuration of the adaptive repair mechanism, including the bandit policy, the exploration parameter ϵ, and the repair operators considered during the search.

The experiments were conducted on a computer equipped with an AMD Ryzen AI 9 HX 370 processor with Radeon 890M graphics, operating at 2.00 GHz, and 32 GB of RAM (31.1 GB usable), under a 64-bit operating system with an x64-based architecture.

### 6.3. Performance of BHGS

#### 6.3.1. Tabu Adaptive Repair

In the following paragraphs, the impact on solution quality and the convergence characteristics of the repair operators based on the Tabu Search technique are analyzed.

##### Solution Quality Analysis

Table 9 reports the BHGS results obtained when solving the SCP, which show competitive performance across the 65 evaluated instances. The algorithm reached the known optimum in 60 instances, equivalent to a 92.31% success rate. Analyzing the median solution cost over the 31 independent runs per instance, the optimum was reached in 45 instances, which provides a stricter evaluation of the typical behavior of the method.

The average $RPD_{best}$ was approximately 0.086%, while the average $RPD_{median}$ was 0.48%, indicating that, even when considering the central performance of the runs, the method remains close to the known optima. The largest median deviations were observed in NRF.5, with $RPD_{median}=7.69\%$, followed by NRH.3, NRH.1, and NRG.4, which shows that these instances present greater difficulty from the stability perspective.

In terms of consistency, the average CV was close to 0.42%, and 19 instances obtained a CV equal to zero, showing identical results in all runs. Overall, the method reaches optimal or near optimal solutions in most cases, although the NRG and NRH groups concentrate the main difficulties. Therefore, the combination of $RPD_{best}$, $RPD_{median}$, and CV allows us to conclude that the proposed approach not only shows a high capacity to achieve good results but also stable behavior across a large part of the benchmark.

**Figure 3:**
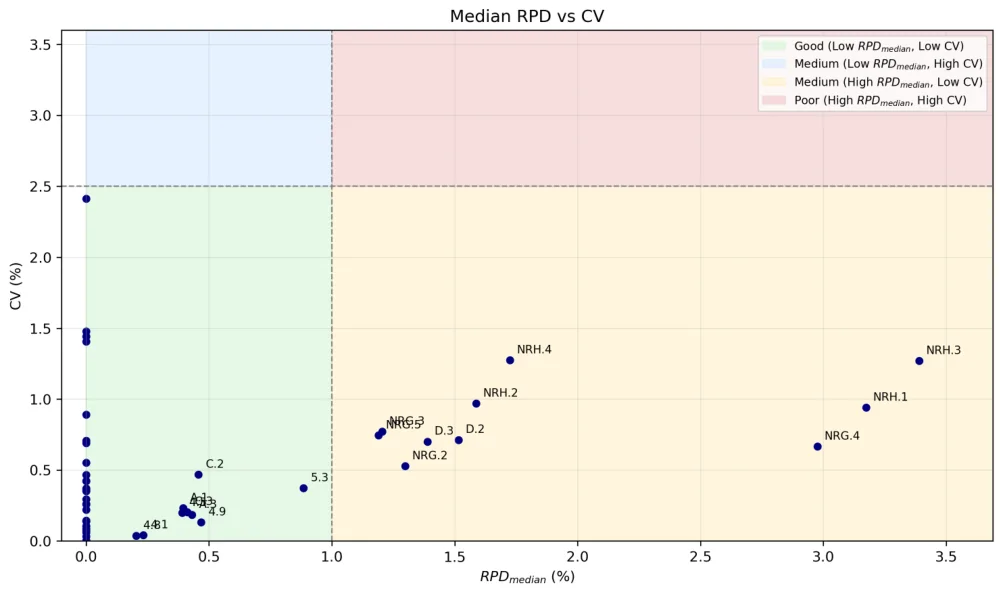
$RPD_{median}$ versus CV for the evaluated SCP benchmark instances. The shaded regions classify the results according to median solution quality and stability across 31 independent runs. The instance NRF.5 was excluded from the plot to improve visualization, since it presents the highest $RPD_{median}$ value.

To evaluate the robustness of the Tabu Search-based repair strategy, Figure 3 jointly analyzes ${\mathrm{RPD}}_{\mathrm{median}}$ and CV across the evaluated SCP benchmark instances, capturing the relationship between median solution quality and stability over the independent runs.

**Figure 4:**
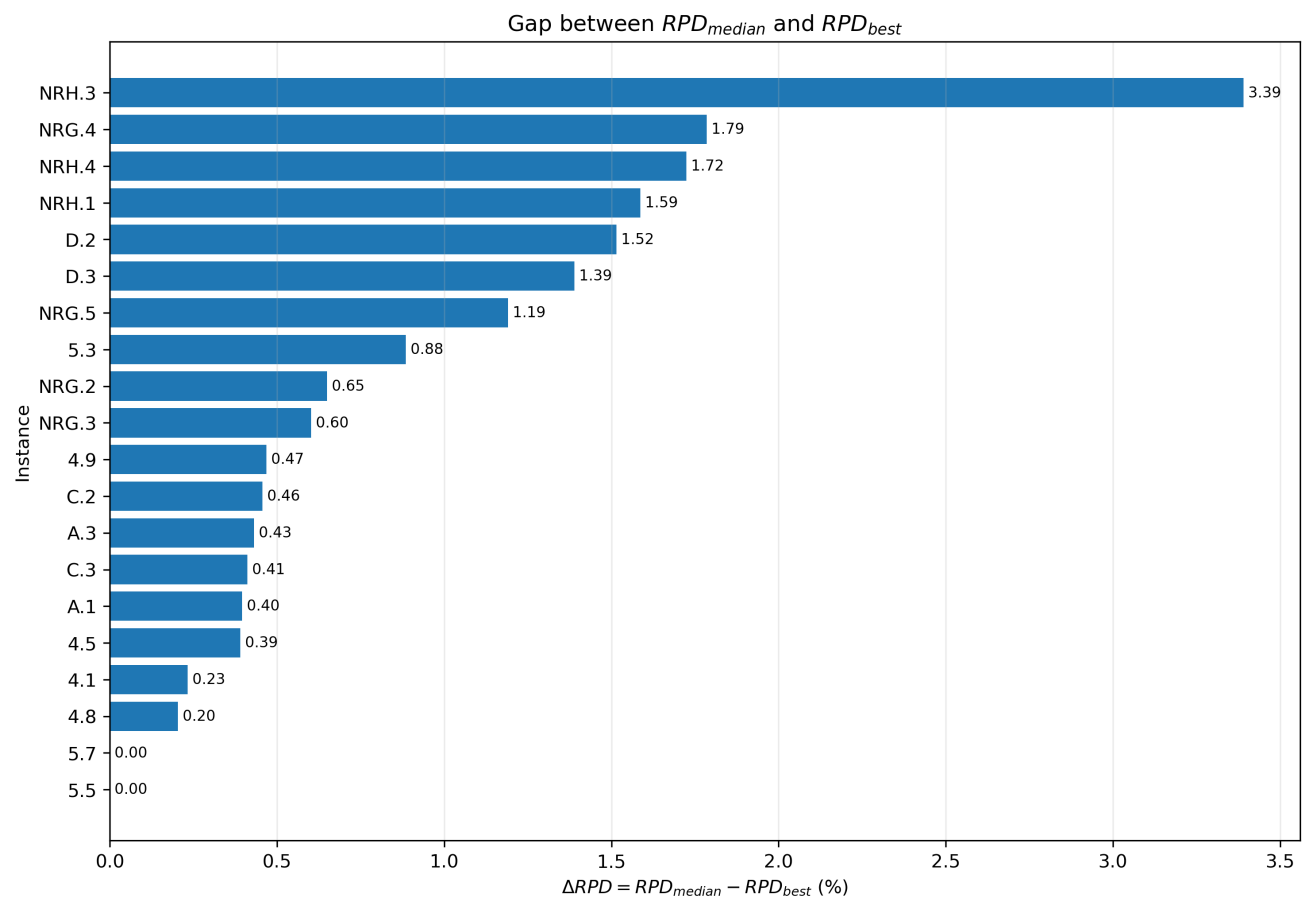
Difference between $RPD_{median}$ and $RPD_{best}$ for the evaluated SCP benchmark instances. The instance NRF.5 was excluded from the plot to improve visualization since it presents the largest gap between $RPD_{median}$ and $RPD_{best}$.

Figure 4 complements this analysis by showing the difference between $RPD_{median}$ and $RPD_{best}$. The largest gaps are observed in NRF.5, NRH.3, NRG.4, NRH.4, and NRH.1, indicating that in these instances the best result obtained does not fully represent the typical behavior of the 31 executions. Consequently, the joint use of $RPD_{best}$, $RPD_{median}$, and CV makes it possible to distinguish between the algorithm’s ability to reach optimal solutions and its regularity in reproducing them consistently.

##### Repair Operator Impact Analysis

Table 10 reports the number of optimal hits achieved by each repair operator for each SCP instance over 31 independent runs. These values do not represent the number of times an operator was selected but rather the number of times it was associated with reaching the known optimum. Therefore, rows with zero values for all three operators indicate that no direct contribution to the known optimum was observed for that instance. This does not imply that the operators were not used or that they did not produce intermediate improvements; instead, it indicates that such improvements did not lead directly to the known optimum under the adopted recording criterion.

Table 11 shows the instance-level selection frequency of each repair operator. The values indicate how often each operator was activated during the search, but they do not necessarily reflect its effectiveness in reaching optimal solutions. In this sense, a high number of selections only indicates frequent use, not a direct contribution to the best-known or optimal solution. This distinction is important because the operator that is most frequently selected is not always the operator that produces the highest number of optimal hits. Consequently, the selection frequency analysis should be considered complementary to the optimal hit analysis.

##### Convergence Analysis

Table 12 summarizes the average exploration and exploitation behavior of BHGS by instance family over 31 iterations. The results show a clear predominance of exploitation across all families, with Avg. XPT values above 93% in all cases, while Avg. XPL remains below 7%. In addition, the average convergence iteration (Avg. CI) indicates that BHGS converges rapidly, since most families stabilize well before the maximum number of iterations. Families B, NRE, and NRF show particularly early convergence, whereas NRG and NRH require more iterations to stabilize, with Avg. CI values of 18.57 and 14.10, respectively. The results show the need to improve the balance between exploration and exploitation, and although the high exploitation rate allows BHGS to converge rapidly, the low levels of exploration limit the ability of the method to escape from promising but suboptimal regions.

**Figure 5:**
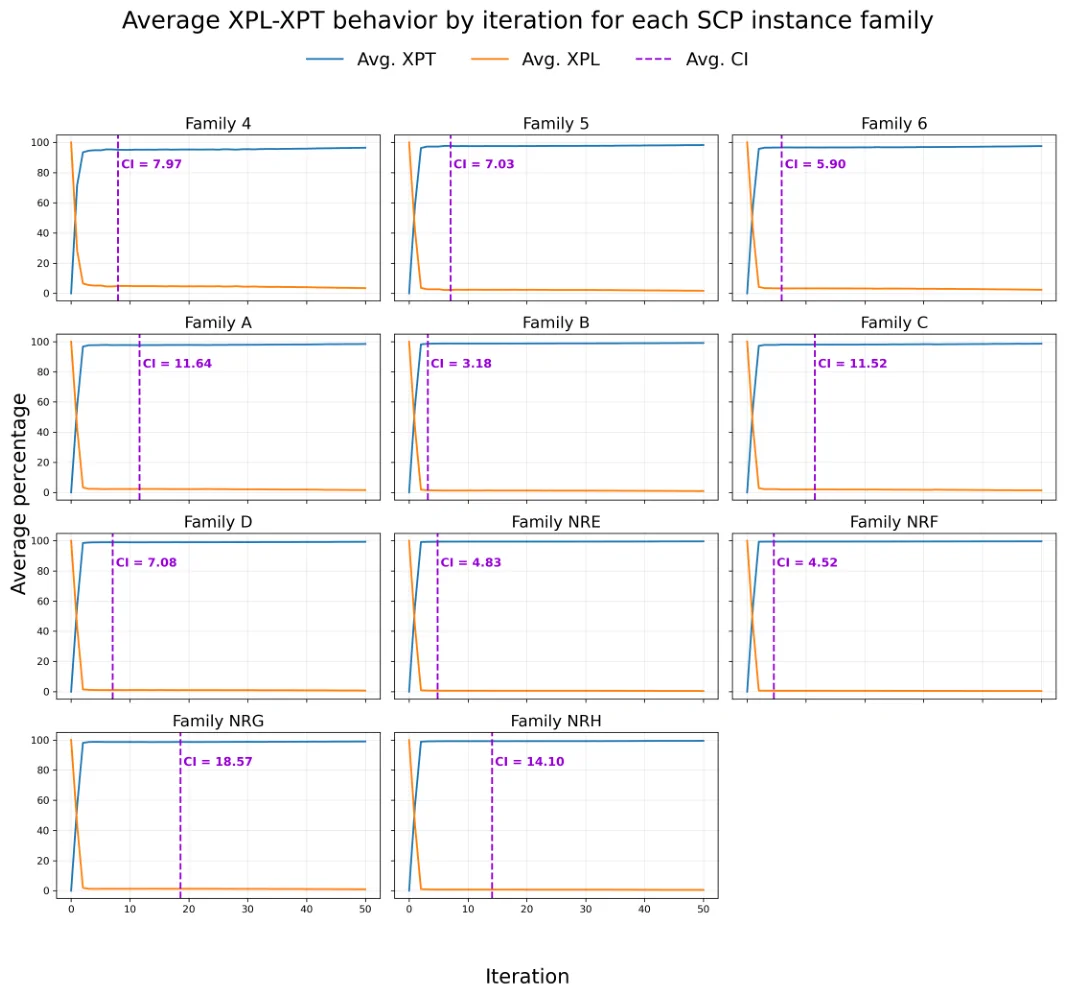
Average XPL-XPT behavior by iteration for each SCP instance family. The dashed vertical line indicates the average convergence iteration (CI).

Figure 5 complements this analysis, as it allows the evolution of XPL and XPT throughout the iterations to be observed. In particular, it shows the rapid predominance of exploitation and the low contribution of exploration during the search process. This behavior reinforces the need to address the convergence behavior of the algorithm, especially to avoid early stabilization in promising but potentially suboptimal regions.

##### Scalability Analysis

The experimental evaluation also provides an initial indication of the behavior of BHGS as the size of the SCP instances increases. The benchmark set includes instances with up to 1000 rows and 10,000 columns. On these larger instances, particularly those belonging to the NRG and NRH families, the proposed method continued to obtain solutions close to the reference values. However, these families also exhibited substantially higher execution times, reaching approximately 52–60 min on average, together with larger differences between the best and median solutions in some instances.

These results indicate that increasing problem size introduces additional computational and stability challenges. Therefore, the current experiments support the applicability of the proposed method within the range of instance sizes considered in this study, but they do not provide sufficient evidence to claim scalability beyond this range. A more comprehensive scalability assessment will require experiments on larger SCP instances, including a specific analysis of the relationship between problem size, solution quality, stability, convergence behavior, and computational cost.

#### 6.3.2. Complex Repair Operators

In the following paragraphs, the impact on solution quality and the convergence characteristics of the repair operators based on the complex technique are analyzed.

##### Quality Solution Analysis

Table 13 shows the results obtained with the repair operators complex_stochastic, complex_penalized, and complex_stochastic_drop, maintaining a good capacity to reach high-quality solutions. Considering the best value obtained per instance, the method reached the known optimum in 58 out of 65 instances, equivalent to an 89.23% success rate according to the known optimum. The median reached the optimum in only 9 out of 65 instances, which indicates that the best result obtained does not necessarily represent the typical behavior of the method. This is reinforced by an average $RPD_{median}$ of approximately 2.03%, higher than the average $RPD_{best}$, which was close to 0.14%.

The average CV was approximately 2.90%. This value indicates that the method presents variability across executions, especially in certain instance families. The largest median deviations are concentrated in the NRF, NRG, and NRH families, where the most difficult cases appear. In particular, the largest median deviations were observed in NRF.3 and NRF.4, both with $RPD_{median}=7.14\%$, followed by NRH.1 with $RPD_{median}=6.35\%$, NRH.5 with $RPD_{median}=5.45\%$, and NRG.4 with $RPD_{median}=5.36\%$. Additionally, execution times for instances 6.4, 5.7, C.1, NRF.4, and NRG.5 are notably higher than the rest of their family, likely due to system interference rather than intrinsic computational cost, as their solution-quality indicators remain consistent with neighboring instances.

**Figure 6:**
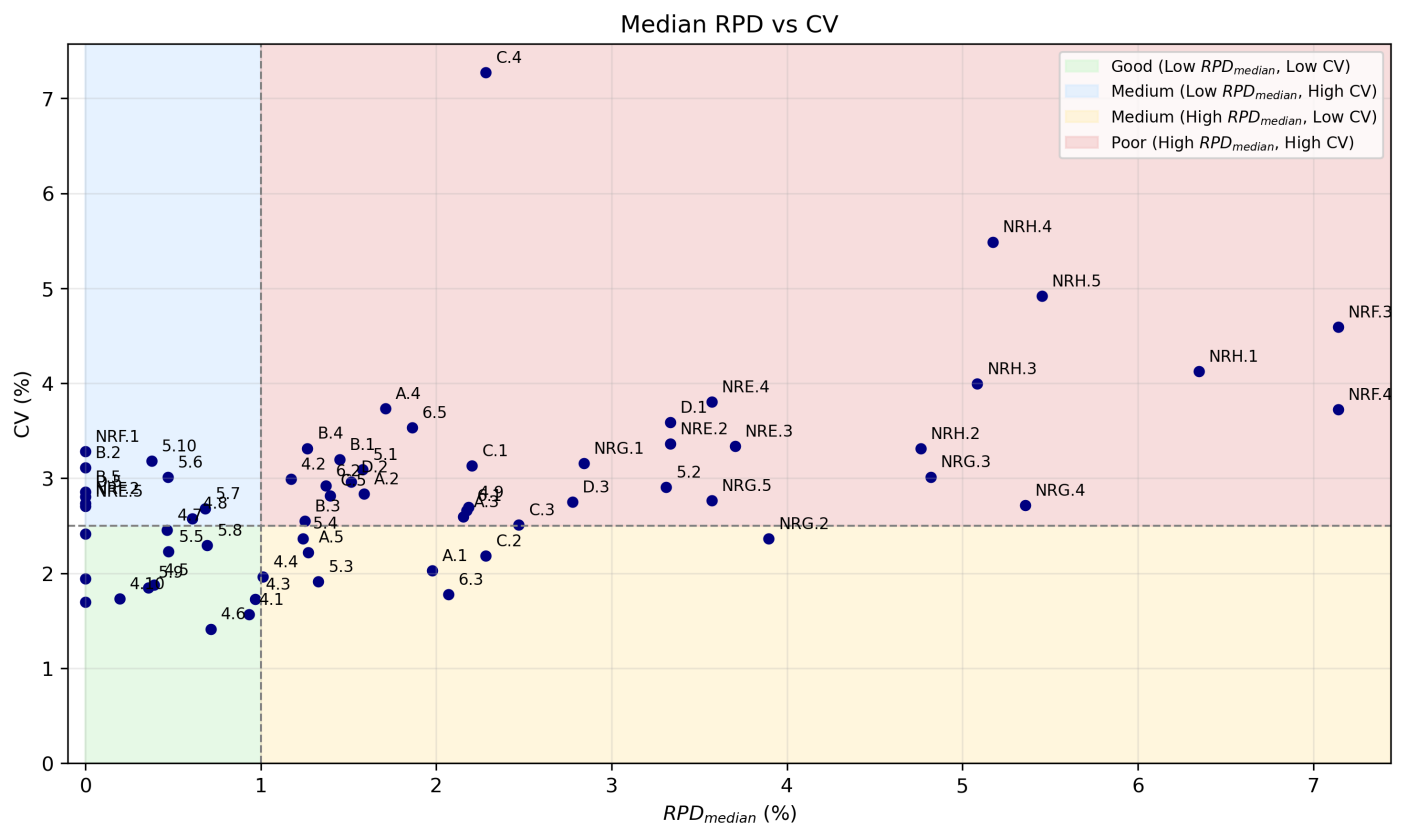
$RPD_{median}$ versus CV for the evaluated SCP benchmark instances. The shaded regions classify the results according to median solution quality and stability across 31 independent runs for operators complex_stochastic, complex_penalized, and complex_stochastic_drop.

Figure 6 reveals a clear dispersion of the instances away from the ${\mathrm{RPD}}_{median}=0$ axis. Only a small number of cases reach median performance equal to the known optimum, while most instances show positive ${\mathrm{RPD}}_{median}$ values. This behavior indicates that the complex repair operators can occasionally produce high-quality solutions, but their median performance is not consistently optimal across the benchmark. The presence of several points with both higher ${\mathrm{RPD}}_{median}$ and higher CV also suggests that, in the most difficult instances, the method loses both solution quality and stability.

**Figure 7:**
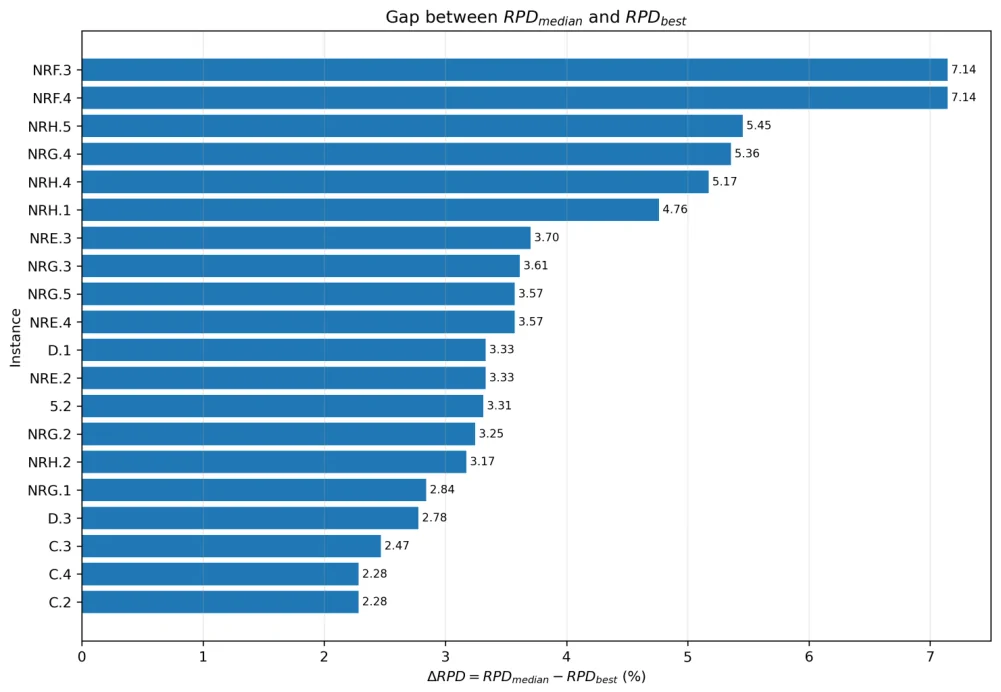
Difference between $RPD_{median}$ and $RPD_{best}$ for the evaluated SCP benchmark instances for operators complex_stochastic, complex_penalized, and complex_stochastic_drop.

Figure 7 shows that the largest gaps between median RPD and best RPD are concentrated in the NRF and NRH families. The highest differences appear in NRF.3 and NRF.4, both with 7.14%, followed by NRH.5, NRG.4, NRH.4, and NRH.1. This indicates that, in these instances, BHGS can obtain high-quality solutions in some runs, but it does not reproduce them consistently. Therefore, the best result provides an optimistic view of the method, while the median reveals lower robustness in the most difficult cases.

##### Repair Operator Complex Impact Analysis

As in Table 10, Table 14 presents the optimal hits results of the complex repair operators. The results demonstrate the direct contribution of each operator for each SCP instance. The results confirm that CSD is the most influential operator since it obtains the highest number of optimal hits in most cases.

Table 15 reports the selection frequency of each complex repair operator for every SCP instance. This analysis allows us to observe how often each operator was activated during the search. However, these values should not be interpreted as a direct indicator of solution quality. In fact, when compared with the optimal hits results, the table shows that the most frequently selected operator is not always the one that contributes most directly to reaching the known optimum. Therefore, this table complements the optimal hits analysis by distinguishing operator usage from operator effectiveness.

##### Convergence Analysis

Table 16 summarizes the average exploration and exploitation behavior of BHGS by SCP family. The table reports the mean and standard deviation of XPL and XPT, together with the convergence iteration CI. The results show a clear predominance of exploitation in all families, with Avg. XPT values above 87% in every case. In addition, the Avg. CI values indicate that several families converge before completing all iterations, although the larger CI values observed in families 4, C, NRG, and NRH suggest slower stabilization in those cases.

**Figure 8:**
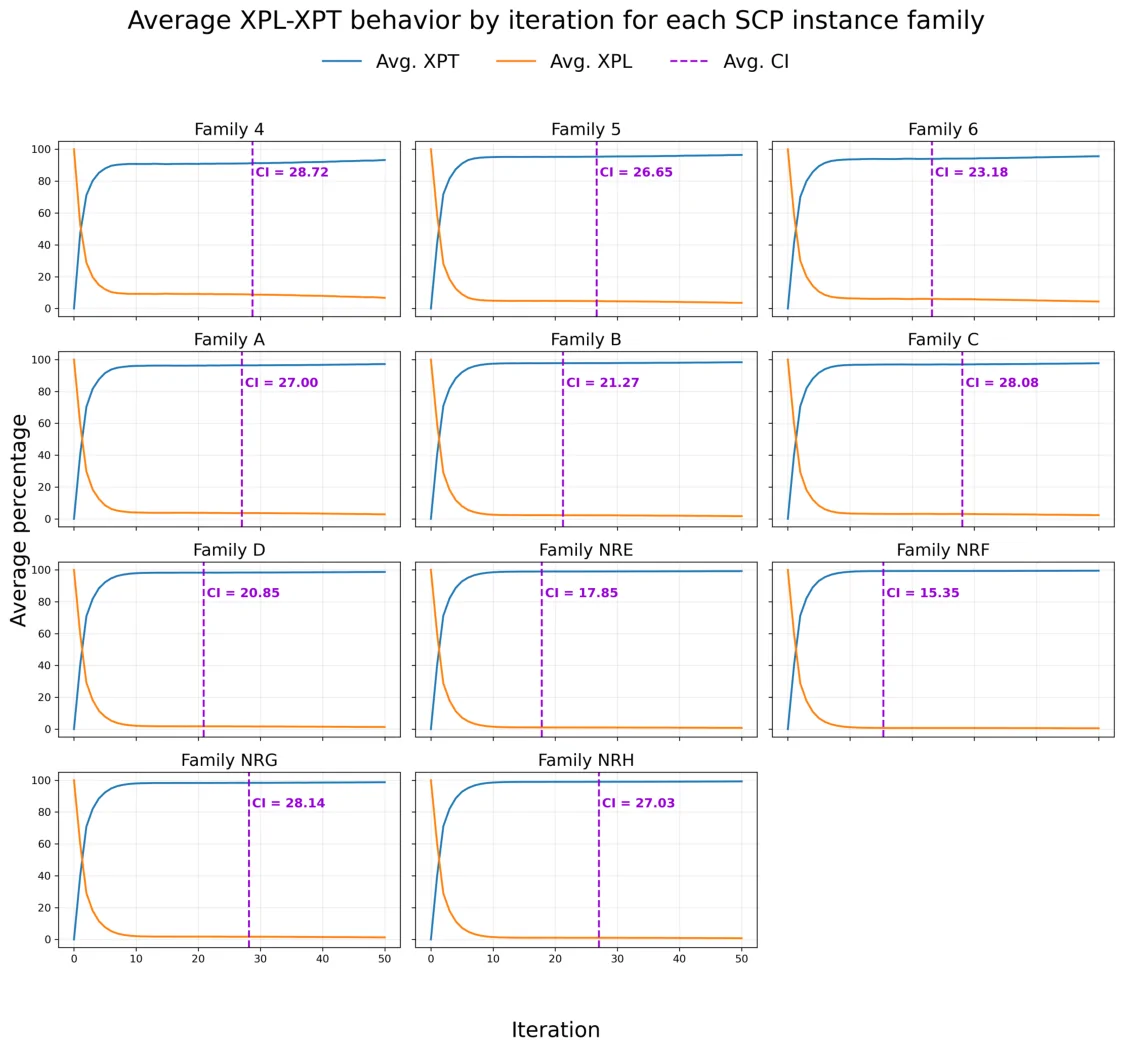
Average XPL-XPT behavior by iteration for each SCP instance family. The dashed vertical line indicates the average convergence iteration (CI).

Figure 8 complements the detailed values reported in Table 16. The plots reinforce the same pattern across all SCP families: XPT rapidly becomes dominant, while XPL decreases sharply during the first iterations and remains low for the rest of the search. The vertical CI line shows that most families converge before the maximum number of iterations, although families 4, C, NRG, and NRH require more iterations to stabilize.

##### Comparative Analysis

Table 17 compares the global behavior of the complex and Tabu Search repair operators in terms of selection frequency, relative contribution to improvement, and ability to reach known optima. These measures allow distinguishing between how frequently an operator is selected and how effectively it contributes to the search process.

For the complex operators, CSD shows the strongest contribution, accounting for 37.21% of the improvement and reaching 1263 known optima, despite CP having the highest selection frequency. In contrast, the Tabu Search operators exhibit a more balanced distribution of improvement, ranging from 32.12% to 34.09%, with TCSD achieving the highest number of optimal hits.

The hit rate values also favor the Tabu Search operators overall. TCSD and TCS achieve rates of 1.90% and 1.36%, respectively, while CSD reaches 1.31% and the remaining complex operators remain below 0.2%. These results suggest that complex relies more strongly on CSD, whereas Tabu Search distributes the contribution more evenly among its operators.

**Figure 9:**
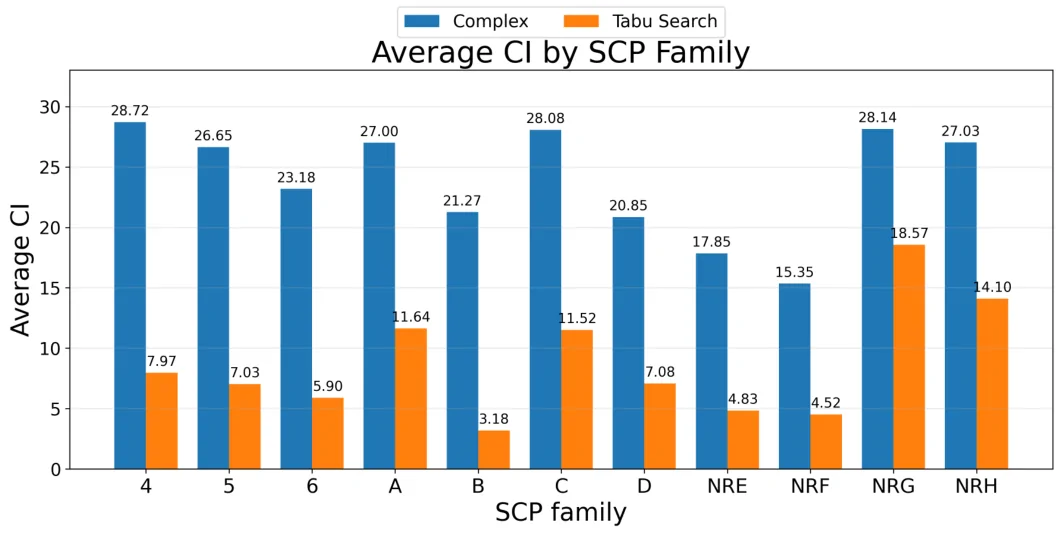
Average CI by SCP family for complex and Tabu Search repair operators.

Figure 9 visually reinforces the convergence pattern reported in Table 18. The complex operators show higher CI values across all SCP families, indicating a later stabilization of the search. In contrast, Tabu Search reaches its final best solution earlier, reflecting a more intensive search dynamic.

Table 19 summarizes the global comparison between the two repair strategies. Tabu Search outperforms the complex operators across all reported indicators except execution time. Convergence is markedly faster under Tabu Search, with Avg. CI = 8.76 compared with 24.01 for the complex operators, and solution quality is consistently better, both in the best case, with Avg. ${\mathrm{RPD}}_{best}$ = 0.086 compared with 0.138, and, more notably, in the typical case, with Avg. ${\mathrm{RPD}}_{median}$ = 0.477 compared with 2.028.

Stability follows the same pattern, with Tabu Search achieving a substantially lower Avg. CV, 0.417 compared with 2.896. This difference is most evident in the median optima count: Tabu Search reaches the known optimum in 45 of 65 instances, compared with only 9 of 65 for the complex operators. This indicates that its advantage is not limited to isolated best-case runs but reflects consistently better behavior across independent executions.

### 6.4. Statistical Analysis

Before applying the paired statistical comparison, the normality assumption was evaluated using the Shapiro Wilk test [26]. Since the Wilcoxon signed rank test compares paired observations, the test was applied to the paired differences between the complex and Tabu Search repair operators. Table 20 shows that the normality assumption was rejected for all evaluated indicators. Therefore, a non-parametric paired test was adopted.

Unlike the six pairwise algorithm comparisons summarized in the comparative analysis, where a Holm correction was applied because all six tests share the same null hypothesis (equal performance of BHGS relative to each competing algorithm across the same instance set), the 65 instance-level tests reported in Table 21 evaluate a distinct hypothesis for each individual SCP instance. Each test compares the paired distribution of fitness values obtained by the two repair strategies on a given, independent problem instance; consequently, these tests are not treated as a family of simultaneous hypotheses about a single overall effect, and no correction for multiple comparisons was applied. This distinction follows standard practice in comparative metaheuristic studies, where per-instance statistical tests are reported as descriptive evidence of consistency across the benchmark rather than as a single inferential claim [27,28].

Table 21 confirms that the superiority of Tabu Search is consistent at the instance level. Tabu Search obtains more wins than losses in 64 out of 65 instances, while complex does not dominate in any instance. In addition, 59 out of 65 instances present statistically significant differences in favor of Tabu Search.

The few non-significant cases, namely D.4, D.5, NRE.1, NRE.5, NRF.2, and NRF.5, are mainly characterized by a high number of ties, meaning that both strategies often reach the same RPD value. Therefore, these cases do not provide evidence against Tabu Search. Instead, they indicate that both methods behave similarly in those particular instances.

The advantage of Tabu Search is especially relevant in the more difficult families, such as NRG and NRH, where all instances show significant differences in its favor. This supports the conclusion that the proposed Tabu Search repair operators improve the typical performance of BHGS across the benchmark, rather than only improving aggregate averages.

#### Effect Size Analysis

Effect size measures were used to complement the comparison between the Tabu-based and complex repair configurations. While statistical tests indicate whether differences are statistically significant, effect size measures quantify the magnitude and practical relevance of those differences [27,28,29,30]. The columns of Table 22 are defined as follows:

- Inst: identifies the SCP instance evaluated in the experiment.
- Family: indicates the instance family to which the corresponding SCP instance belongs.
- Tabu Median: reports the median fitness value obtained by BHGS using the Tabu-based repair configuration across the independent runs of the corresponding instance. Since the SCP is a minimization problem, lower values indicate better performance.
- Complex Median: reports the median fitness value obtained by BHGS using the complex repair configuration across the independent runs of the corresponding instance.
- $A_{12}$: corresponds to the Vargha Delaney effect size measure. In this study, values greater than 0.5 indicate that the Tabu-based configuration tends to obtain better fitness values than the complex configuration, values close to 0.5 indicate similar behavior, and values below 0.5 favor the complex configuration.
- Cliff’s δ: represents Cliff’s delta effect size measure, which quantifies the dominance of one configuration over the other. Positive values indicate that the Tabu-based configuration tends to outperform the complex configuration, while negative values indicate the opposite.
- Magnitude: classifies the strength of the effect size according to the absolute value of Cliff’s δ. The possible categories are negligible, small, medium, and large.
- Favors: indicates which repair configuration is favored by the effect size analysis for each instance.

### 6.5. Comparison with State-of-the-Art Metaheuristics

This subsection performs a comparative analysis between the proposed BHGS and six other metaheuristics previously applied to the SCP: the Sine Cosine Algorithm (SCA) [31], the Pendulum Search Algorithm (PSA) [32], the Gray Wolf Optimizer (GWO) [33], the Binary Growth Optimizer (BGO) [34], the Binary Dream Optimization Algorithm (BDOA) [19], and the Binary Arithmetic Optimization Algorithm (BAOA) [25].

The comparative results for the reference algorithms were obtained from their respective cited publications and were not generated through new reimplementations in this study. In particular, PSA, SCA, GWO, BAOA, and BGO were originally evaluated under the experimental frameworks defined in their corresponding publications, which are not necessarily identical to the experimental framework adopted for BHGS. Therefore, the purpose of this comparison is not to claim a controlled head-to-head evaluation under an identical experimental protocol but rather to assess the relative solution quality reported by the different algorithmic frameworks on the same OR-Library benchmark instances.

Although the complete experimental evaluation of BHGS was conducted on 65 OR-Library benchmark instances, the comparison with the six reference algorithms was restricted to a common subset of 45 instances, since published results for all seven algorithms were available only for these instances. Consequently, all comparative indicators and statistical analyzes reported in this subsection are computed exclusively over this common set of 45 instances using the corresponding reported results.

Table 23 summarizes the comparison in terms of average RPD, percentage of instances reaching the known optimum, and average rank. On the common set of 45 instances, BHGS and BDOA achieve average RPD values close to zero (0.009% and 0.023%, respectively) and reach the known optimum in 97.8% and 93.3% of the compared instances, respectively. Their average ranks (1.89 and 1.93) also indicate similar overall performance. The remaining algorithms (PSA, SCA, GWO, BAOA, and BGO) obtain substantially higher average ranks, ranging from 4.64 to 5.09, with BAOA achieving the lowest average RPD within this group.

Table 24 shows that the Friedman test confirms highly significant global differences among the seven algorithms: $\chi^{2}=169.11$, df=6, $p\approx 6.93\times 10^{-34}$, well below the 0.05 threshold. This rejects the null hypothesis of equivalent performance and justifies the post hoc analysis.

Table 25 shows that the Wilcoxon post hoc test confirms that BHGS and BDOA are statistically indistinguishable from each other (p=0.180), supporting that both approaches based on adaptive bandit-driven repair achieve equivalent top-tier performance on the SCP. In contrast, BHGS significantly outperforms the remaining five algorithms (BAOA, SCA, PSA, GWO, BGO), all with p<0.001 after Holm correction. This confirms that BHGS’s superiority over this group is not explained by random variation across instances.

**Figure 10:**
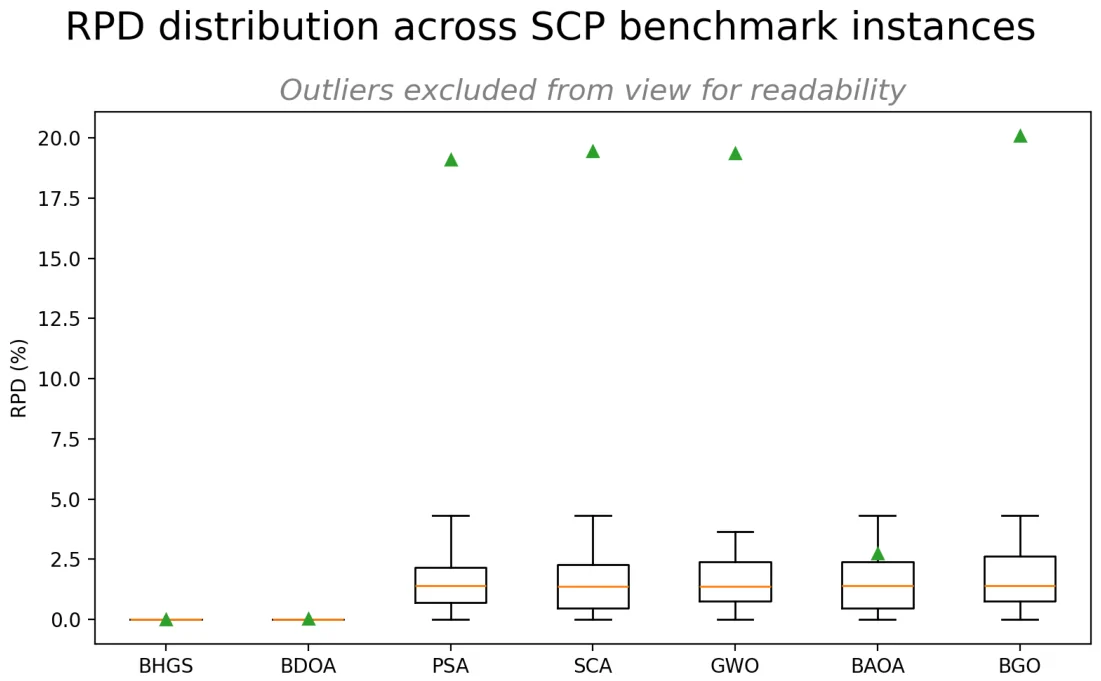
RPD distribution across the SCP benchmark instances for BHGS and the compared metaheuristics (outliers excluded from view for readability). The green triangle marks the mean, the orange line marks the median.

**Figure 11:**
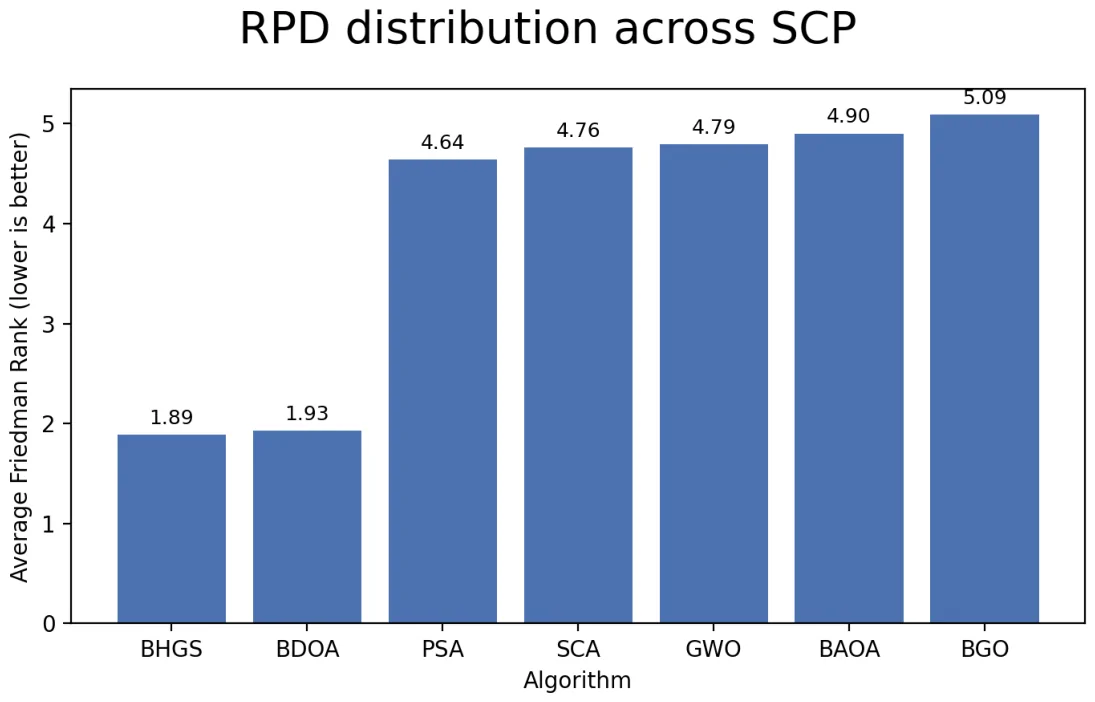
Average Friedman ranking of BHGS and the compared metaheuristics across the SCP benchmark instances (lower is better).

Figure 10 and Figure 11 complement Table 23. Figure 10 shows the full RPD distribution per algorithm, revealing the spread and the case of BAOA (mean well above its median). Figure 11 shows the average ranking from the same table as a bar chart, for an at-a-glance comparison of the seven algorithms.

## 7. Novelty and Contributions

Previous binary adaptations of continuous metaheuristics have largely concentrated on the binarization stage, particularly on how continuous solutions are transformed into binary representations. In the present work, we investigate a complementary aspect of the binary adaptation process: the restoration of feasibility after binarization.

The main contribution of this study is therefore not the introduction of the adaptive repair selection mechanism itself, which follows the data-driven repair principle previously explored in [19], but the experimental demonstration that the feasibility repair strategy can materially affect the performance of the resulting binary metaheuristic, even when the binarization and adaptive selection mechanisms remain unchanged.

To isolate this effect, the HGS search dynamics, the V3-ELIT binarization scheme, the adaptive repair selection mechanism, the benchmark instances, and the experimental conditions are kept fixed, while the feasibility restoration strategy is varied. In particular, complex repair operators are compared with Tabu Search-based repair operators under the same BHGS framework. This controlled design allows the effect of the repair mechanism itself to be separated from the effects of binarization and adaptive operator selection.

The experimental results show that the Tabu Search-based repairers provide better solution quality and variability indicators than the complex repairers under the evaluated configurations. Since the remaining components of the framework are unchanged, these differences provide evidence that improving the feasibility-restoration stage can enhance the performance of the binary search process without modifying the binarization strategy.

This finding shifts part of the design focus in binary metaheuristics from binarization alone toward the interaction between binarization and feasibility restoration. Binarization determines how a continuous solution is mapped into the binary domain, whereas the repair mechanism determines how an infeasible binary solution is transformed into a feasible one. The results obtained in this study indicate that the latter stage should not be treated merely as a corrective procedure but as an active component capable of influencing subsequent search dynamics and final solution quality.

The comparison with BDOA should be interpreted within this context. BHGS does not statistically outperform BDOA on the common benchmark subset; instead, both approaches achieve comparable performance. Therefore, the contribution of BHGS is not based on superiority over BDOA but on providing additional evidence that the effectiveness of a binary metaheuristic depends not only on the continuous to binary transformation but also on the quality of the feasibility restoration mechanism coupled with the search process.

## 8. Conclusions

This paper investigated the influence of feasibility repair strategies within a Binary Hunger Games Search framework for the Set Covering Problem. The proposed approach combines the continuous search dynamics of Hunger Games Search with the V3-ELIT binarization scheme, an adaptive operator selection mechanism, and two families of feasibility restoration operators: complex and Tabu Search-based repairers. This study was designed not only to obtain feasible solutions but also to determine whether changes in the repair strategy produce measurable differences in solution quality, variability, search behavior, and computational cost.

The experimental results show that the repair strategy has a significant influence on the behavior of the binary metaheuristic. Under the same Hunger Games Search mechanism, V3-ELIT binarization scheme, adaptive selector, benchmark instances, and experimental conditions, the Tabu Search-based repairers consistently produced better solution-quality and variability indicators than the complex repairers. They also reached their best-observed solutions earlier during the search for the evaluated instance families. Since the remaining algorithmic components were kept unchanged, these differences provide direct evidence that the feasibility-restoration strategy is not an interchangeable component of the binary adaptation.

The complete experimental evaluation of BHGS was conducted on 65 benchmark instances. Across this full benchmark set, BHGS reached the corresponding reference value in 60 of the 65 instances, representing 92.31%, and obtained an average $RPD_{best}$ of 0.086%. These results characterize the performance of the proposed method over the complete set of instances considered in the experimental study.

A separate state-of-the-art comparison was conducted on a subset of 45 benchmark instances. This subset was used because comparable results for all seven algorithms considered in the comparison were available only for these common instances. Therefore, the comparative indicators reported in Table 23 refer exclusively to this 45-instance subset and should not be interpreted as results obtained over the complete 65-instance experimental set. On these 45 common instances, BHGS achieved an average RPD of 0.009%, reached the known optimum in 44 of the 45 instances (97.8%), and obtained the best average rank, with a value of 1.89. The closest competitor, BDOA, obtained an average RPD of 0.023%, reached the known optimum in 42 of the 45 instances (93.3%), and achieved an average rank of 1.93. The remaining methods exhibited substantially larger average RPD values, ranging from 2.756% for BAOA to more than 19% for PSA, SCA, GWO, and BGO. These results show that BHGS maintains a highly competitive level of solution quality within the common subset for which a direct comparison among all seven algorithms was possible.

The statistical analysis of the repair strategies supports the experimental observations. Since the normality assumption was rejected, the comparisons were performed using the Wilcoxon signed-rank test. Statistically significant differences were found in solution quality and variability, while the corresponding effect-size analysis indicated that the magnitude of these differences was also relevant. Therefore, the advantage observed for the Tabu Search-based repairers is supported not only by descriptive performance measures but also by the statistical comparison between the evaluated configurations.

The influence of the repair operator is not restricted to the immediate recovery of feasibility. Once an infeasible binary solution has been repaired, its objective function value participates in the update of the best and worst solutions, hunger levels, adaptive weights, and subsequent population movements of Hunger Games Search. Changes introduced during restoration can therefore propagate to later stages of the optimization process. This interaction helps explain why replacing the repair strategy, while keeping the binarization and adaptive-selection mechanisms unchanged, can lead to measurable differences in the final performance of the algorithm.

The improvements obtained with the Tabu Search-based repairers, however, require additional computational effort. Complex repairers showed lower execution times, whereas the Tabu Search-based variants provided better typical solution quality, lower variability, and earlier attainment of their best-observed solutions. The results therefore reveal a clear trade-off between computational cost and search effectiveness, rather than an unconditional superiority of one repair family across all performance criteria.

Overall, the results indicate that feasibility restoration should be regarded as an integral part of the search process when adapting a continuous metaheuristic to the Set Covering Problem. The fact that the Hunger Games Search dynamics, binarization strategy, and adaptive operator-selection mechanism remain unchanged while different repair families produce statistically supported performance differences shows that restoration is capable of materially altering the behavior of the resulting binary algorithm. The complete evaluation of 65 benchmark instances characterizes the overall performance of BHGS, whereas the separate comparison of the 45 common instances provides a direct assessment against the six reference algorithms for which comparable results were available. Together, these findings support the effectiveness of the proposed BHGS configuration and motivate a broader investigation of feasibility restoration mechanisms alongside binarization strategies in constrained binary optimization.

## 9. Future Works

Future research should further investigate the internal behavior of feasibility repair operators and their interaction with the search dynamics of Binary Hunger Games Search. In particular, it would be relevant to analyze how the decisions introduced during repair affect the fitness values subsequently used to update hunger levels, adaptive weights, and population movements.

A first research direction is the evaluation of additional memory-based repair mechanisms. This includes different Tabu tenures, aspiration criteria, dynamic memory sizes, frequency-based memory, and alternative neighborhood structures. Such experiments would help determine which components of Tabu Search are responsible for the observed improvements and whether simpler configurations can preserve solution quality while reducing computational costs.

A second direction is to perform ablation studies on the proposed repair operators. The contribution of feasibility restoration, column removal, local repair, Tabu memory, and adaptive operator selection should be evaluated separately. This would make it possible to determine whether the improvements are mainly produced by the local search procedure, the memory mechanism, the adaptive selector, or the interaction among these components.

Future studies should also analyze the behavior of the multi-armed bandit controller in greater detail. Relevant aspects include operator-selection frequencies, accumulated rewards, the evolution of estimated operator values, and the relationship between operator performance and instance characteristics. Alternative credit-assignment rules and selection policies could also be evaluated to determine whether the controller can react more effectively to changes in the search state.

Another relevant direction is the development of adaptive activation criteria for the repair and Tabu Search procedures. Instead of applying the same repair intensity whenever an infeasible solution is generated, future approaches could adjust the neighborhood size, Tabu tenure, or local-search effort according to the degree of infeasibility, stagnation level, iteration stage, or recent improvement history.

Finally, the proposed repair strategies should be evaluated on larger SCP instances and related constrained binary optimization problems. This would allow a more rigorous assessment of their scalability, generality, and practical limitations, particularly regarding the trade-off between solution quality, stability, convergence, and computational cost.

## Figures and Tables

**Table 1:** Coverage of seven demand zones by candidate hospital locations.

Candidate Hospital	Zone 1	Zone 2	Zone 3	Zone 4	Zone 5	Zone 6	Zone 7	Cost
$H_{1}$	1	1	0	1	0	0	0	120
$H_{2}$	0	1	1	0	1	0	0	100
$H_{3}$	1	0	0	1	1	1	0	150
$H_{4}$	0	0	1	0	0	1	1	90
$H_{5}$	0	1	0	0	1	0	1	110

**Table 2:** Main stages of the original Hunger Games Search algorithm.

Stage	Description
Stage 1: Initialization	Initializes the main parameters, the population of individuals, and the hunger values. Each individual represents a candidate solution in the continuous search space.
Stage 2: Fitness Evaluation	The fitness of all individuals is calculated. Then, the best fitness value BF, the worst fitness value WF, the best individual $X_{b}$, and the best individual index BI are updated.
Stage 3: Hunger Update	The hunger value of each individual is updated according to its relative fitness. The best individual receives a hunger value equal to zero, while the remaining individuals update their hunger level based on the hunger mechanism.
Stage 4: Adaptive Weight Update	The adaptive weights $W_{1}$ and $W_{2}$ are computed from the hunger values. These weights regulate the influence of the best individual and the movement amplitude during the search.
Stage 5: Position Update	Each individual updates its position according to the HGS movement rules. This stage contains two search behaviors: exploration and exploitation.
Stage 5.1: Exploration Phase	When the exploration condition is satisfied, the individual updates its position by applying a random perturbation around its current location. This phase promotes diversification of the search process.
Stage 5.2: Exploitation Phase	When the exploitation condition is satisfied, the individual updates its position using the best solution $X_{b}$, the adaptive weights, and the stochastic movement factor *R*. This phase intensifies the search around promising regions.
Stage 6: Iteration Update	The iteration counter is increased, and the algorithm repeats the evaluation, hunger update, weight update, and position update stages until the maximum number of iterations is reached.
Stage 7: Output	After the stopping criterion is met, the algorithm returns the best fitness value BF and the best solution $X_{b}$.

**Table 3:** Feasibility repair operators used in the adaptive repair selection mechanism.

Repair Operator	Description	Mathematical Formulation
tabu_complex_stochastic	Stochastic cost–coverage repair. One column is randomly selected from the five candidates with the lowest cost–coverage ratios before applying the common Tabu Search refinement.	$\rho_{j}=\frac{c_{j}}{q_{j}}$
tabu_complex_penalized	Frequency-penalized cost–coverage repair. Frequently selected columns receive a progressively higher effective cost before applying the common Tabu Search refinement.	$\rho_{j}^{P}=\frac{c_{j}\left(1+0.2p_{j}\right)}{q_{j}}$
tabu_complex_stochastic_drop	Stochastic cost–coverage repair followed by the removal of redundant columns, prioritizing higher-cost columns, before applying the common Tabu Search refinement.	$\sum_{k\neq j}a_{ik}x_{k}\geq 1,\;\forall i:a_{ij}=1$

**Table 4:** Variables used in the Binary HGS with Adaptive Repair Selection algorithm.

Variable	Description
*N*	Population size, i.e., the number of individuals used by HGS.
*D*	Dimension of the continuous search space. In the SCP, this corresponds to the number of available subsets or columns.
*i*	Index of an individual in the population, with i=1,…,N.
*j*	Index of a decision variable, subset, or column of the SCP, with j=1,…,D.
*k*	Index of a repair operator in the repair pool, with k=1,…,K.
*t*	Current iteration of the algorithm, with t=1,…,T.
*T*	Maximum number of iterations of the algorithm.
$X_{i}$	Continuous position of individual *i* generated and updated by the original HGS movement rules.
$B_{i}$	Binary solution obtained after applying the V3-ELIT binarization scheme to the continuous vector $X_{i}$.
${\hat{B}}_{i}$	Feasible binary solution obtained after applying a repair operator to $B_{i}$ when the binarized solution is infeasible.
$B^{best}$	Best feasible binary solution found during the search.
BF	Best fitness value associated with $B^{best}$. For the SCP, this value corresponds to the minimum cost found so far.
R	Set of available repair operators used by the adaptive repair selection mechanism.
$R_{k}$	Repair operator *k* belonging to the repair set R.
$R_{t}$	Repair operator selected at iteration *t* by the multi-armed bandit mechanism.
$Q(R_{k})$	Estimated utility of repair operator $R_{k}$, computed from the rewards observed after its application.
$N(R_{k})$	Number of times that repair operator $R_{k}$ has been selected during the search.

**Table 5:** Description of the OR-Library SCP benchmark sets, including number of instances, problem size, cost range, density, and optimal solution status data taken from [25].

Instance Family	Number of Instances	m	n	Cost Range	Density (%)	Optimal Solution
4	10	200	1000	[1100]	2.00	known
5	10	200	2000	[1100]	2.00	known
6	5	200	1000	[1100]	5.00	known
A	5	300	3000	[1100]	2.00	known
B	5	300	3000	[1100]	5.00	known
C	5	400	4000	[1100]	2.00	known
D	5	400	4000	[1100]	5.00	known
NRE	5	500	5000	[1100]	10.00	known
NRF	5	500	5000	[1100]	20.00	known
NRG	5	1000	10,000	[1100]	2.00	BKS (not proven)
NRH	5	1000	10,000	[1100]	5.00	BKS (not proven)

Note: for the NRG and NRH families, the reference values correspond to Best Known Solutions (BKS), as optimality has not been formally proven for these instances.

**Table 6:** Interpretation criteria for ΔRPD.

Category	Criterion	Interpretation
High consistency	ΔRPD=0%	The best and median performances coincide. The best solution is representative of the typical behavior of the algorithm.
Low gap	0<ΔRPD≤1%	There is a small difference between the best result and the median behavior, indicating good central performance.
Moderate gap	1%<ΔRPD≤3%	The best result does not fully represent the typical behavior of the algorithm, suggesting moderate variability in solution quality.
High gap	ΔRPD>3%	The best result is likely associated with occasional successful runs, while the median indicates a more distant central behavior from the optimum.

**Table 7:** Presetting analysis for the ϵ parameter.

ϵ	Instances	Avg. ${\mathrm{RPD}}_{\mathrm{best}}$	Avg. ${\mathrm{RPD}}_{\mathrm{median}}$	Avg. CV	Avg. Time	Best Optima	Media Optima
0.05	18	0.101000	0.303000	0.371000	414.959000	16/18	14/18
0.10	18	0.088000	0.303000	0.368000	373.181000	17/18	14/18
0.15	18	0.088000	0.215000	0.264000	716.079000	17/18	15/18
0.20	18	0.088000	0.272000	0.393000	411.591000	17/18	14/18
0.30	18	0.101000	0.296000	0.354000	370.986000	16/18	13/18

**Table 8:** Parameter values used in the proposed BHGS with adaptive repair selection.

Parameter	Value
Population size	300
Maximum iterations	50
Independent runs	31
$l_{\max}$	0.9
$l_{\min}$	0.1
LH	100
Numerical stability constant	$10^{-12}$
Fitness scaling constant	$10^{3}$
Binarization scheme	V3 ELIT
Adaptive selection scheme	Bandit-based selection
Bandit policy	Epsilon greedy
ϵ	0.15
Complex repair operators	complex_stochastic_drop
	complex_stochastic
	complex_penalized
Tabu Search repair operators	tabu_complex_stochastic_drop
	tabu_complex_stochastic
	tabu_complex_penalized

**Table 9:** Experimental results obtained by the proposed Binary HGS with Adaptive Repair Selection with Tabu Operators.

Inst	Opt	Best	Median	Avg.	Std.	${\mathrm{RPD}}_{\mathrm{best}}$	${\mathrm{RPD}}_{\mathrm{median}}$	CV	Avg. Time
4.1	429	429	430.00	429.97	0.18	0.00	0.23	0.04%	0:03:23
4.2	512	512	512.00	512.00	0.00	0.00	0.00	0.00%	0:03:24
4.3	516	516	516.00	516.00	0.00	0.00	0.00	0.00%	0:03:18
4.4	494	494	494.00	494.23	0.43	0.00	0.00	0.09%	0:03:26
4.5	512	512	514.00	513.03	1.02	0.00	0.39	0.20%	0:03:24
4.6	560	560	560.00	560.03	0.18	0.00	0.00	0.03%	0:03:23
4.7	430	430	430.00	430.19	0.60	0.00	0.00	0.14%	0:03:22
4.8	492	492	493.00	492.97	0.18	0.00	0.20	0.04%	0:03:14
4.9	641	641	644.00	643.94	0.85	0.00	0.47	0.13%	0:03:29
4.10	514	514	514.00	514.00	0.00	0.00	0.00	0.00%	0:03:20
5.1	253	253	253.00	253.00	0.00	0.00	0.00	0.00%	0:04:30
5.2	302	302	302.00	302.61	1.28	0.00	0.00	0.42%	0:04:42
5.3	226	226	228.00	227.55	0.85	0.00	0.88	0.37%	0:04:31
5.4	242	242	242.00	242.03	0.18	0.00	0.00	0.07%	0:04:27
5.5	211	211	211.00	211.10	0.30	0.00	0.00	0.14%	0:04:34
5.6	213	213	213.00	213.00	0.00	0.00	0.00	0.00%	0:04:30
5.7	293	293	293.00	293.03	0.18	0.00	0.00	0.06%	0:04:32
5.8	288	288	288.00	288.00	0.00	0.00	0.00	0.00%	0:04:34
5.9	279	279	279.00	279.19	0.40	0.00	0.00	0.14%	0:04:32
5.10	265	265	265.00	265.00	0.00	0.00	0.00	0.00%	0:04:31
6.1	138	138	138.00	138.06	0.36	0.00	0.00	0.26%	0:03:16
6.2	146	146	146.00	146.00	0.00	0.00	0.00	0.00%	0:03:15
6.3	145	145	145.00	145.10	0.54	0.00	0.00	0.37%	0:03:12
6.4	131	131	131.00	131.00	0.00	0.00	0.00	0.00%	0:03:09
6.5	161	161	161.00	161.19	0.75	0.00	0.00	0.46%	0:03:16
A.1	253	253	254.00	254.29	0.59	0.00	0.40	0.23%	0:06:17
A.2	252	252	252.00	252.42	0.89	0.00	0.00	0.35%	0:06:17
A.3	232	232	233.00	232.87	0.43	0.00	0.43	0.18%	0:06:18
A.4	234	234	234.00	234.26	0.51	0.00	0.00	0.22%	0:06:18
A.5	236	236	236.00	236.06	0.25	0.00	0.00	0.11%	0:06:19
B.1	69	69	69.00	69.06	0.25	0.00	0.00	0.36%	0:06:05
B.2	76	76	76.00	76.00	0.00	0.00	0.00	0.00%	0:06:06
B.3	80	80	80.00	80.00	0.00	0.00	0.00	0.00%	0:06:04
B.4	79	79	79.00	79.00	0.00	0.00	0.00	0.00%	0:06:13
B.5	72	72	72.00	72.00	0.00	0.00	0.00	0.00%	0:06:10
C.1	227	227	227.00	227.52	0.96	0.00	0.00	0.42%	0:08:19
C.2	219	219	220.00	220.06	1.03	0.00	0.46	0.47%	0:08:26
C.3	243	243	244.00	244.13	0.50	0.00	0.41	0.20%	0:08:18
C.4	219	219	219.00	219.84	1.21	0.00	0.00	0.55%	0:08:25
C.5	215	215	215.00	215.39	0.56	0.00	0.00	0.26%	0:08:23
D.1	60	60	60.00	60.23	0.43	0.00	0.00	0.71%	0:08:14
D.2	66	66	67.00	66.68	0.48	0.00	1.52	0.71%	0:08:06
D.3	72	72	73.00	72.52	0.51	0.00	1.39	0.70%	0:08:14
D.4	62	62	62.00	62.00	0.00	0.00	0.00	0.00%	0:08:07
D.5	61	61	61.00	61.03	0.18	0.00	0.00	0.29%	0:08:06
NRE.1	29	29	29.00	29.00	0.00	0.00	0.00	0.00%	0:11:17
NRE.2	30	30	30.00	30.23	0.43	0.00	0.00	1.41%	0:11:23
NRE.3	27	27	27.00	27.19	0.40	0.00	0.00	1.48%	0:11:39
NRE.4	28	28	28.00	28.06	0.25	0.00	0.00	0.89%	0:11:23
NRE.5	28	28	28.00	28.00	0.00	0.00	0.00	0.00%	0:11:30
NRF.1	14	14	14.00	14.00	0.00	0.00	0.00	0.00%	0:13:11
NRF.2	15	15	15.00	15.00	0.00	0.00	0.00	0.00%	0:13:28
NRF.3	14	14	14.00	14.13	0.34	0.00	0.00	2.41%	0:13:05
NRF.4	14	14	14.00	14.00	0.00	0.00	0.00	0.00%	0:13:25
NRF.5	13	13	14.00	13.81	0.40	0.00	7.69	2.91%	0:14:09
NRG.1	176	176	176.00	176.90	1.22	0.00	0.00	0.69%	0:52:23
NRG.2	154	155	156.00	156.29	0.82	0.65	1.30	0.53%	0:52:54
NRG.3	166	167	168.00	168.68	1.30	0.60	1.20	0.77%	0:52:47
NRG.4	168	170	173.00	172.94	1.15	1.19	2.98	0.67%	0:52:38
NRG.5	168	168	170.00	169.45	1.26	0.00	1.19	0.74%	0:52:34
NRH.1	63	64	65.00	64.65	0.61	1.59	3.17	0.94%	0:57:43
NRH.2	63	64	64.00	64.52	0.63	1.59	1.59	0.97%	0:59:43
NRH.3	59	59	61.00	61.00	0.77	0.00	3.39	1.27%	0:59:15
NRH.4	58	58	59.00	59.03	0.75	0.00	1.72	1.27%	0:59:06
NRH.5	55	55	55.00	55.35	0.80	0.00	0.00	1.44%	0:56:41

**Table 10:** Instance level optimal hits achieved by the Tabu Search-based repair operators.

Instance	TCS-D	TCS	TCP	Instance	TCS-D	TCS	TCP
4.1	**1**	0	0	B.4	**13**	12	6
4.2	**19**	7	5	B.5	**14**	13	2
4.3	10	**13**	8	C.1	**11**	7	4
4.4	**18**	5	1	C.2	**10**	1	2
4.5	**13**	0	2	C.3	**1**	0	0
4.6	**20**	8	2	C.4	6	**7**	6
4.7	**15**	9	4	C.5	**14**	2	4
4.8	**1**	0	0	D.1	**11**	9	4
4.9	**2**	0	0	D.2	4	**5**	1
4.10	**22**	6	3	D.3	**9**	3	3
5.1	**16**	10	5	D.4	**17**	12	2
5.2	**12**	9	4	D.5	**15**	11	4
5.3	**6**	1	0	NRE.1	**12**	**12**	6
5.4	**14**	12	4	NRE.2	**10**	8	6
5.5	**18**	5	5	NRE.3	**13**	9	3
5.6	11	**12**	8	NRE.4	11	**14**	4
5.7	**22**	7	1	NRE.5	**17**	9	5
5.8	**19**	8	4	NRF.1	**14**	11	6
5.9	**17**	5	3	NRF.2	11	**14**	6
5.10	**19**	6	6	NRF.3	8	**13**	6
6.1	11	**12**	7	NRF.4	**16**	12	3
6.2	**16**	13	2	NRF.5	**4**	1	1
6.3	12	**14**	4	NRG.1	**13**	4	1
6.4	12	**15**	4	NRG.2	0	0	0
6.5	**15**	7	7	NRG.3	0	0	0
A.1	**1**	**1**	0	NRG.4	0	0	0
A.2	**13**	9	2	NRG.5	**6**	3	1
A.3	**3**	0	2	NRH.1	0	0	0
A.4	**12**	10	2	NRH.2	0	0	0
A.5	**14**	9	6	NRH.3	**1**	0	0
B.1	12	**15**	2	NRH.4	**6**	1	0
B.2	14	**15**	2	NRH.5	**15**	4	6
B.3	13	**14**	4				

TCS-D denotes tabu_complex_stochastic_drop, TCS denotes tabu_complex_stochastic, and TCP denotes tabu_complex_penalized. Values correspond to the number of optimal hits obtained by each repair operator for each SCP instance. Bold values indicate the highest number of optimal hits for each instance.

**Table 11:** Number of selections by instance achieved by each repair operator.

Instance	TCS-D	TCS	TCP	Instance	TCS-D	TCS	TCP
4.1	**854**	430	235	B.4	370	**575**	574
4.2	493	**534**	492	B.5	**623**	474	422
4.3	**541**	528	450	C.1	484	501	**534**
4.4	**670**	505	344	C.2	**693**	535	291
4.5	**673**	442	404	C.3	**601**	426	492
4.6	481	**680**	358	C.4	**578**	468	473
4.7	526	**665**	328	C.5	**693**	529	297
4.8	500	**715**	304	D.1	475	**550**	494
4.9	**566**	545	408	D.2	473	518	**528**
4.10	437	**734**	348	D.3	440	**630**	449
5.1	**672**	479	368	D.4	554	**581**	384
5.2	**641**	500	378	D.5	456	**582**	481
5.3	**687**	505	327	NRE.1	524	**573**	422
5.4	**636**	532	351	NRE.2	472	**619**	428
5.5	**646**	424	449	NRE.3	**778**	379	362
5.6	365	**691**	463	NRE.4	504	**611**	404
5.7	**816**	491	212	NRE.5	**648**	406	465
5.8	**783**	515	221	NRF.1	535	**579**	405
5.9	570	**582**	367	NRF.2	537	**641**	341
5.10	548	**620**	351	NRF.3	441	**580**	498
6.1	534	**564**	421	NRF.4	**601**	494	424
6.2	**720**	500	299	NRF.5	**730**	487	302
6.3	509	**561**	449	NRG.1	542	265	**712**
6.4	**557**	541	421	NRG.2	**572**	419	528
6.5	**647**	396	476	NRG.3	**630**	452	437
A.1	286	586	**647**	NRG.4	419	516	**584**
A.2	493	**518**	508	NRG.5	489	375	**655**
A.3	**654**	443	422	NRH.1	418	370	**731**
A.4	456	**631**	432	NRH.2	**648**	430	441
A.5	**651**	501	367	NRH.3	**545**	442	532
B.1	**681**	357	481	NRH.4	**714**	351	454
B.2	461	483	**575**	NRH.5	**577**	365	**577**
B.3	506	**560**	453				

TCS-D denotes tabu_complex_stochastic_drop, TCS denotes tabu_complex_stochastic, and TCP denotes tabu_complex_penalized. Values correspond to the number of times each repair operator was selected for each SCP instance. Bold values indicate the most frequently selected operator for each instance.

**Table 12:** XPL-XPT analysis by instance family.

Family	Instances	Avg. XPL	Std. XPL	Avg. XPT	Std. XPT	Avg. CI	Std. CI
4	10	6.79	1.74	93.21	1.74	7.97	7.76
5	10	4.93	0.88	95.07	0.88	7.03	7.13
6	5	5.70	0.80	94.30	0.80	5.90	7.57
A	5	4.81	0.79	95.19	0.79	11.64	10.60
B	5	3.92	0.32	96.08	0.32	3.18	2.07
C	5	4.53	0.72	95.47	0.72	11.52	9.59
D	5	3.73	0.29	96.27	0.29	7.08	9.22
NRE	5	3.36	0.10	96.64	0.10	4.83	6.67
NRF	5	3.31	0.06	96.69	0.06	4.52	7.95
NRG	5	4.02	0.58	95.98	0.58	18.57	13.37
NRH	5	3.52	0.20	96.48	0.20	14.10	13.47

**Table 13:** Experimental results obtained by the proposed Binary HGS with Adaptive Repair Selection with Complex Operators.

Inst	Opt	Best	Median	Avg.	Std.	${\mathrm{RPD}}_{\mathrm{best}}$	${\mathrm{RPD}}_{\mathrm{median}}$	CV	Avg. Time
4.1	429	429	433	434.98	6.82	0.00	0.93	1.57%	0:00:52
4.2	512	512	518	522.95	15.65	0.00	1.17	2.99%	0:00:59
4.3	516	516	521	524.31	9.06	0.00	0.97	1.73%	0:01:07
4.4	494	494	499	502.05	9.86	0.00	1.01	1.96%	0:01:07
4.5	512	512	514	518.40	9.73	0.00	0.39	1.88%	0:01:09
4.6	560	560	564	566.76	8.01	0.00	0.71	1.41%	0:01:03
4.7	430	430	432	436.14	10.71	0.00	0.47	2.45%	0:01:07
4.8	492	492	495	500.40	12.90	0.00	0.61	2.58%	0:01:03
4.9	641	641	655	661.23	17.82	0.00	2.18	2.69%	0:01:04
4.10	514	514	515	520.04	9.00	0.00	0.19	1.73%	0:00:51
5.1	253	253	257	260.87	8.07	0.00	1.58	3.09%	0:01:48
5.2	302	302	312	313.18	9.10	0.00	3.31	2.91%	0:02:09
5.3	226	226	229	230.43	4.41	0.00	1.33	1.91%	0:01:35
5.4	242	242	245	246.30	5.83	0.00	1.24	2.37%	0:01:35
5.5	211	211	212	214.69	4.78	0.00	0.47	2.23%	0:01:35
5.6	213	213	214	217.56	6.55	0.00	0.47	3.01%	0:01:35
5.7	293	293	295	298.71	8.01	0.00	0.68	2.68%	0:15:27
5.8	288	288	290	292.87	6.72	0.00	0.69	2.29%	0:01:37
5.9	279	279	280	282.74	5.23	0.00	0.36	1.85%	0:01:42
5.10	265	265	266	269.63	8.58	0.00	0.38	3.18%	0:02:12
6.1	138	138	141	141.59	3.77	0.00	2.17	2.66%	0:00:52
6.2	146	146	148	149.62	4.37	0.00	1.37	2.92%	0:00:51
6.3	145	145	148	147.30	2.62	0.00	2.07	1.78%	0:00:52
6.4	131	131	131	132.69	2.26	0.00	0.00	1.70%	0:30:57
6.5	161	161	164	165.13	5.84	0.00	1.86	3.54%	0:00:49
A.1	253	254	258	259.24	5.26	0.40	1.98	2.03%	0:02:41
A.2	252	252	256	258.74	7.34	0.00	1.59	2.84%	0:03:28
A.3	232	232	237	239.19	6.20	0.00	2.16	2.59%	0:02:20
A.4	234	234	238	241.51	9.02	0.00	1.71	3.74%	0:02:14
A.5	236	236	239	240.46	5.34	0.00	1.27	2.22%	0:02:16
B.1	69	69	70	70.52	2.25	0.00	1.45	3.20%	0:02:10
B.2	76	76	76	77.33	2.41	0.00	0.00	3.11%	0:03:13
B.3	80	80	81	81.75	2.08	0.00	1.25	2.55%	0:02:46
B.4	79	79	80	80.97	2.68	0.00	1.27	3.32%	0:02:24
B.5	72	72	72	73.02	2.09	0.00	0.00	2.86%	0:02:23
C.1	227	227	232	233.89	7.33	0.00	2.20	3.13%	0:25:02
C.2	219	219	224	225.32	4.92	0.00	2.28	2.18%	0:04:08
C.3	243	243	249	250.46	6.29	0.00	2.47	2.51%	0:06:02
C.4	219	219	224	227.83	16.57	0.00	2.28	7.27%	0:03:56
C.5	215	215	218	220.33	6.21	0.00	1.40	2.82%	0:04:25
D.1	60	60	62	62.14	2.23	0.00	3.33	3.59%	0:04:04
D.2	66	66	67	67.77	2.01	0.00	1.52	2.96%	0:04:13
D.3	72	72	74	74.34	2.05	0.00	2.78	2.75%	0:04:16
D.4	62	62	62	62.78	1.52	0.00	0.00	2.42%	0:04:23
D.5	61	61	61	61.91	1.74	0.00	0.00	2.80%	0:04:21
NRE.1	29	29	29	29.26	0.57	0.00	0.00	1.95%	0:06:17
NRE.2	30	30	31	31.42	1.06	0.00	3.33	3.36%	0:06:20
NRE.3	27	27	28	28.22	0.94	0.00	3.70	3.34%	0:06:01
NRE.4	28	28	29	29.00	1.10	0.00	3.57	3.80%	0:05:19
NRE.5	28	28	28	28.31	0.77	0.00	0.00	2.70%	0:06:19
NRF.1	14	14	14	14.32	0.47	0.00	0.00	3.28%	0:06:49
NRF.2	15	15	15	15.18	0.42	0.00	0.00	2.74%	0:05:37
NRF.3	14	14	15	14.81	0.68	0.00	7.14	4.59%	0:12:12
NRF.4	14	14	15	14.55	0.54	0.00	7.14	3.73%	0:43:18
NRF.5	13	13	14	14.12	0.46	0.00	7.69	3.28%	0:06:56
NRG.1	176	176	181	181.81	5.74	0.00	2.84	3.16%	0:34:15
NRG.2	154	155	160	160.54	3.80	0.65	3.90	2.37%	0:34:19
NRG.3	166	168	174	174.98	5.27	1.20	4.82	3.01%	0:33:40
NRG.4	168	168	177	177.68	4.83	0.00	5.36	2.72%	0:35:08
NRG.5	168	168	174	175.29	4.85	0.00	3.57	2.76%	1:49:10
NRH.1	63	64	67	67.27	2.77	1.59	6.35	4.12%	0:38:54
NRH.2	63	64	66	66.66	2.21	1.59	4.76	3.31%	0:37:10
NRH.3	59	61	62	63.14	2.52	3.39	5.08	3.99%	0:36:26
NRH.4	58	58	61	61.61	3.38	0.00	5.17	5.49%	0:47:15
NRH.5	55	55	58	58.02	2.86	0.00	5.45	4.92%	0:49:53

**Table 14:** Instance level optimal hits achieved by the complex repair operators.

Instance	CSD	CS	CP	Instance	CSD	CS	CP
4.1	**3**	0	0	B.4	**37**	0	1
4.2	**20**	0	0	B.5	**53**	2	7
4.3	**21**	0	0	C.1	**7**	0	0
4.4	**18**	0	1	C.2	**2**	0	0
4.5	**8**	0	0	C.3	**1**	0	0
4.6	**14**	0	0	C.4	**9**	0	1
4.7	**31**	0	0	C.5	**11**	0	1
4.8	**6**	0	0	D.1	**24**	0	0
4.9	**1**	0	0	D.2	**9**	0	2
4.10	**40**	1	0	D.3	**14**	0	0
5.1	**10**	0	0	D.4	**56**	2	4
5.2	**12**	0	0	D.5	**48**	1	11
5.3	**7**	0	0	NRE.1	**59**	6	7
5.4	**23**	0	0	NRE.2	**12**	2	2
5.5	**16**	0	0	NRE.3	**10**	0	3
5.6	**43**	1	0	NRE.4	**23**	3	5
5.7	**10**	0	0	NRE.5	**54**	9	14
5.8	**39**	0	0	NRF.1	**47**	6	10
5.9	**10**	0	0	NRF.2	**54**	10	13
5.10	**42**	2	0	NRF.3	**26**	3	2
6.1	**28**	1	3	NRF.4	**29**	8	7
6.2	**11**	2	1	NRF.5	**3**	1	0
6.3	**37**	1	2	NRG.1	**11**	0	0
6.4	**36**	6	6	NRG.2	0	0	0
6.5	**30**	2	7	NRG.3	0	0	0
A.1	0	0	0	NRG.4	**1**	0	0
A.2	**15**	0	0	NRG.5	**3**	0	0
A.3	**2**	0	0	NRH.1	0	0	0
A.4	**4**	0	0	NRH.2	0	0	0
A.5	**7**	0	0	NRH.3	0	0	0
B.1	**29**	4	7	NRH.4	**3**	0	1
B.2	**51**	2	1	NRH.5	**18**	0	0
B.3	**15**	0	0				

CSD denotes complex_stochastic_drop, CS denotes complex_stochastic, and CP denotes complex_penalized. Values correspond to the number of optimal hits obtained by each repair operator for each SCP instance. Bold values indicate the highest number of optimal hits for each instance.

**Table 15:** Selections by instance achieved by each complex repair operator.

Instance	CSD	CS	CP	Instance	CSD	CS	CP
4.1	1481	1467	**1609**	B.4	1343	1404	**1810**
4.2	1474	1439	**1644**	B.5	1152	1477	**1928**
4.3	**2095**	1060	1402	C.1	**1637**	1469	1451
4.4	**1690**	1440	1427	C.2	**1602**	1515	1440
4.5	**2180**	1109	1268	C.3	1574	1318	**1665**
4.6	**1616**	1566	1375	C.4	1255	**1695**	1607
4.7	**2076**	1513	968	C.5	1506	1173	**1878**
4.8	**1586**	1442	1529	D.1	1460	1341	**1756**
4.9	**1630**	1450	1477	D.2	987	1767	**1803**
4.10	**1797**	1255	1505	D.3	1408	1534	**1615**
5.1	1461	1161	**1935**	D.4	1585	**1655**	1317
5.2	1613	1253	**1691**	D.5	1393	**1645**	1519
5.3	**1722**	1381	1454	NRE.1	**1598**	1521	1438
5.4	1493	1519	**1545**	NRE.2	**1655**	1404	1498
5.5	**1780**	1502	1275	NRE.3	1287	1218	**2052**
5.6	**1645**	1434	1478	NRE.4	1385	**1648**	1524
5.7	1494	1219	**1844**	NRE.5	**1583**	1427	1547
5.8	**1726**	1368	1463	NRF.1	1495	**1818**	1244
5.9	**1940**	1286	1331	NRF.2	1329	**1699**	1529
5.10	**1711**	1408	1438	NRF.3	1397	1316	**1844**
6.1	1500	1165	**1892**	NRF.4	1343	1595	**1619**
6.2	1552	1452	**1553**	NRF.5	1373	1366	**1818**
6.3	**1634**	1620	1303	NRG.1	1485	1475	**1597**
6.4	1305	1488	**1764**	NRG.2	1003	1739	**1815**
6.5	**1782**	1339	1436	NRG.3	964	1422	**2171**
A.1	1423	1476	**1658**	NRG.4	1137	1509	**1911**
A.2	1421	**1570**	1566	NRG.5	1177	1620	**1760**
A.3	**1676**	1217	1664	NRH.1	1242	1600	**1715**
A.4	1107	**1775**	1675	NRH.2	1383	**1615**	1559
A.5	1538	1419	**1600**	NRH.3	1023	1742	**1792**
B.1	1252	1428	**1877**	NRH.4	1443	1348	**1766**
B.2	1576	**1660**	1321	NRH.5	1197	1394	**1966**
B.3	1367	1398	**1792**				

CSD denotes complex_stochastic_drop, CS denotes complex_stochastic, and CP denotes complex_penalized. Values correspond to the number of times each repair operator was selected for each SCP instance. Bold values indicate the most frequently selected operator for each instance. Selection frequency should not be interpreted as a direct measure of contribution to the optimum.

**Table 16:** XPL-XPT analysis by instance family.

Family	Instances	Avg. XPL	Std. XPL	Avg. XPT	Std. XPT	Avg. CI	Std. CI
4	10	12.03	4.51	87.97	4.51	28.72	14.58
5	10	8.44	3.04	91.56	3.04	26.65	15.02
6	5	9.62	3.11	90.38	3.11	23.18	14.71
A	5	7.69	2.77	92.31	2.77	27.00	14.08
B	5	6.45	2.34	93.55	2.34	21.27	14.54
C	5	7.10	2.57	92.90	2.57	28.08	14.92
D	5	6.17	2.29	93.83	2.29	20.85	14.16
NRE	5	5.40	2.10	94.60	2.10	17.85	14.29
NRF	5	5.26	2.04	94.74	2.04	15.35	12.78
NRG	5	6.31	2.25	93.69	2.25	28.14	14.86
NRH	5	5.67	2.10	94.33	2.10	27.03	14.76

**Table 17:** Global comparison between complex and Tabu Search repair operators.

Strategy	Operator	Selections	Selection Share (%)	Improvement Share (%)	Optimal Hits	Hit Rate (%)
Complex	CSD	96,744	32.66	37.21	1263	1.31
Complex	CS	94,748	31.99	31.27	75	0.08
Complex	CP	104,713	35.35	31.52	119	0.11
Tabu Search	TCSD	37,024	37.50	33.78	705	1.90
Tabu Search	TCS	33,481	33.91	32.12	454	1.36
Tabu Search	TCP	28,230	28.59	34.09	201	0.71

CSD denotes complex stochastic drop, CS denotes complex stochastic, and CP denotes complex penalized. TCSD denotes Tabu complex stochastic drop, TCS denotes Tabu complex stochastic, and TCP denotes Tabu complex penalized. Selection share measures how often each operator was selected relative to the operators within the same repair strategy. Improvement share represents the relative contribution of each operator to the total improvement achieved within its corresponding repair strategy, while hit rate measures the proportion of selections that resulted in reaching a known optimum.

**Table 18:** Comparison of XPL, XPT, and CI between complex and Tabu Search repair operators.

Family	Complex XPL	Complex XPT	Complex CI	Tabu XPL	Tabu XPT	Tabu CI
4	12.03	87.97	28.72	6.79	93.21	7.97
5	8.44	91.56	26.65	4.93	95.07	7.03
6	9.62	90.38	23.18	5.70	94.30	5.90
A	7.69	92.31	27.00	4.81	95.19	11.64
B	6.45	93.55	21.27	3.92	96.08	3.18
C	7.10	92.90	28.08	4.53	95.47	11.52
D	6.17	93.83	20.85	3.73	96.27	7.08
NRE	5.40	94.60	17.85	3.36	96.64	4.83
NRF	5.26	94.74	15.35	3.31	96.69	4.52
NRG	6.31	93.69	28.14	4.02	95.98	18.57
NRH	5.67	94.33	27.03	3.52	96.48	14.10

**Table 19:** Global comparison between complex and Tabu Search repair operators.

Strategy	Instances	Avg. CI	Avg. ${\mathrm{RPD}}_{\mathrm{best}}$	Avg. ${\mathrm{RPD}}_{\mathrm{median}}$	Avg. CV	Best Optima	Median Optima
Complex	65	24.01	0.138	2.028	2.896	58/65	9/65
Tabu Search	65	8.76	0.086	0.477	0.417	60/65	45/65

**Table 20:** Shapiro Wilk normality test applied separately to complex and Tabu Search results using V3 ELIT.

Strategy	Indicator	W	*p* Value	Decision
Complex	${\mathrm{RPD}}_{best}$	0.3951	$6.66\times 10^{-15}$	Not normal
Complex	${\mathrm{RPD}}_{median}$	0.8011	$5.99\times 10^{-8}$	Not normal
Complex	CV	0.9073	$1.35\times 10^{-4}$	Not normal
Complex	Avg. Time	0.5927	$3.78\times 10^{-12}$	Not normal
Tabu Search	${\mathrm{RPD}}_{best}$	0.2895	$4.09\times 10^{-16}$	Not normal
Tabu Search	${\mathrm{RPD}}_{median}$	0.4584	$4.22\times 10^{-14}$	Not normal
Tabu Search	CV	0.7331	$1.50\times 10^{-9}$	Not normal
Tabu Search	Avg. Time	0.5860	$2.96\times 10^{-12}$	Not normal

**Table 21:** Wilcoxon signed rank test by SCP instance comparing Tabu Search versus complex using V3 ELIT.

Instance	Comparison	Wins	Ties	Losses	*p* Value
4.1	Tabu Search vs. complex	19	11	1	$1.06\times 10^{-4}$
4.2	Tabu Search vs. complex	22	9	0	$3.91\times 10^{-5}$
4.3	Tabu Search vs. complex	21	10	0	$5.60\times 10^{-5}$
4.4	Tabu Search vs. complex	19	8	4	$2.98\times 10^{-4}$
4.5	Tabu Search vs. complex	22	7	2	$1.05\times 10^{-4}$
4.6	Tabu Search vs. complex	25	6	0	$1.18\times 10^{-5}$
4.7	Tabu Search vs. complex	19	12	0	$1.11\times 10^{-4}$
4.8	Tabu Search vs. complex	11	18	2	$3.44\times 10^{-3}$
4.9	Tabu Search vs. complex	27	3	1	$4.92\times 10^{-6}$
4.10	Tabu Search vs. complex	11	20	0	$3.13\times 10^{-3}$
5.1	Tabu Search vs. complex	27	4	0	$5.46\times 10^{-6}$
5.2	Tabu Search vs. complex	24	6	1	$2.22\times 10^{-5}$
5.3	Tabu Search vs. complex	19	10	2	$2.18\times 10^{-3}$
5.4	Tabu Search vs. complex	23	8	0	$2.48\times 10^{-5}$
5.5	Tabu Search vs. complex	21	10	0	$2.25\times 10^{-5}$
5.6	Tabu Search vs. complex	12	19	0	$2.17\times 10^{-3}$
5.7	Tabu Search vs. complex	28	3	0	$3.01\times 10^{-6}$
5.8	Tabu Search vs. complex	12	19	0	$2.16\times 10^{-3}$
5.9	Tabu Search vs. complex	25	5	1	$1.02\times 10^{-5}$
5.10	Tabu Search vs. complex	16	15	0	$3.94\times 10^{-4}$
6.1	Tabu Search vs. complex	18	13	0	$1.84\times 10^{-4}$
6.2	Tabu Search vs. complex	25	6	0	$1.09\times 10^{-5}$
6.3	Tabu Search vs. complex	13	17	1	$2.00\times 10^{-3}$
6.4	Tabu Search vs. complex	9	22	0	$7.00\times 10^{-3}$
6.5	Tabu Search vs. complex	12	17	2	$5.00\times 10^{-3}$
A.1	Tabu Search vs. complex	27	3	1	$6.87\times 10^{-6}$
A.2	Tabu Search vs. complex	21	6	4	$2.29\times 10^{-4}$
A.3	Tabu Search vs. complex	24	5	2	$1.79\times 10^{-5}$
A.4	Tabu Search vs. complex	26	4	1	$7.85\times 10^{-6}$
A.5	Tabu Search vs. complex	28	3	0	$3.37\times 10^{-6}$
B.1	Tabu Search vs. complex	8	22	1	$2.00\times 10^{-2}$
B.2	Tabu Search vs. complex	6	25	0	$2.00\times 10^{-2}$
B.3	Tabu Search vs. complex	25	6	0	$1.31\times 10^{-6}$
B.4	Tabu Search vs. complex	17	14	0	$2.20\times 10^{-4}$
B.5	Tabu Search vs. complex	5	26	0	$2.50\times 10^{-2}$
C.1	Tabu Search vs. complex	23	7	1	$3.39\times 10^{-5}$
C.2	Tabu Search vs. complex	24	5	2	$1.72\times 10^{-5}$
C.3	Tabu Search vs. complex	29	2	0	$2.48\times 10^{-6}$
C.4	Tabu Search vs. complex	25	3	3	$1.88\times 10^{-5}$
C.5	Tabu Search vs. complex	23	6	2	$2.29\times 10^{-5}$
D.1	Tabu Search vs. complex	21	7	3	$1.83\times 10^{-4}$
D.2	Tabu Search vs. complex	11	19	1	$5.00\times 10^{-3}$
D.3	Tabu Search vs. complex	20	7	4	$7.46\times 10^{-4}$
D.4	Tabu Search vs. complex	2	29	0	$1.80\times 10^{-1}$
D.5	Tabu Search vs. complex	6	24	1	$5.30\times 10^{-2}$
NRE.1	Tabu Search vs. complex	1	30	0	$3.17\times 10^{-1}$
NRE.2	Tabu Search vs. complex	17	13	1	$3.46\times 10^{-4}$
NRE.3	Tabu Search vs. complex	19	12	0	$2.01\times 10^{-5}$
NRE.4	Tabu Search vs. complex	13	17	1	$1.00\times 10^{-3}$
NRE.5	Tabu Search vs. complex	0	31	0	1.00
NRF.1	Tabu Search vs. complex	6	25	0	$1.40\times 10^{-2}$
NRF.2	Tabu Search vs. complex	1	30	0	$3.17\times 10^{-1}$
NRF.3	Tabu Search vs. complex	18	12	1	$1.24\times 10^{-4}$
NRF.4	Tabu Search vs. complex	9	22	0	$3.00\times 10^{-3}$
NRF.5	Tabu Search vs. complex	5	25	1	$1.02\times 10^{-1}$
NRG.1	Tabu Search vs. complex	20	7	4	$3.45\times 10^{-4}$
NRG.2	Tabu Search vs. complex	23	4	4	$7.36\times 10^{-5}$
NRG.3	Tabu Search vs. complex	28	1	2	$4.62\times 10^{-6}$
NRG.4	Tabu Search vs. complex	23	4	4	$2.00\times 10^{-4}$
NRG.5	Tabu Search vs. complex	22	5	4	$6.81\times 10^{-5}$
NRH.1	Tabu Search vs. complex	20	11	0	$8.38\times 10^{-5}$
NRH.2	Tabu Search vs. complex	22	6	3	$3.27\times 10^{-4}$
NRH.3	Tabu Search vs. complex	19	8	4	$7.83\times 10^{-4}$
NRH.4	Tabu Search vs. complex	20	6	5	$5.21\times 10^{-4}$
NRH.5	Tabu Search vs. complex	16	13	2	$1.00\times 10^{-3}$

**Table 22:** Effect size analysis between Tabu-based and complex repair configurations.

Inst	Family	Tabu Median	Complex Median	$A_{12}$	Cliff’s δ	Magnitude	Favors
4.1	4	430	434	0.859	0.718	large	Tabu
4.2	4	512	518	0.892	0.785	large	Tabu
4.3	4	516	521	0.887	0.774	large	Tabu
4.4	4	494	499	0.857	0.713	large	Tabu
4.5	4	514	514	0.818	0.636	large	Tabu
4.6	4	560	564	0.921	0.842	large	Tabu
4.7	4	430	432	0.802	0.603	large	Tabu
4.8	4	493	496	0.770	0.540	large	Tabu
4.9	4	644	655	0.950	0.899	large	Tabu
4.10	4	514	515	0.780	0.559	large	Tabu
5.1	5	253	257	0.946	0.892	large	Tabu
5.2	5	302	312	0.907	0.815	large	Tabu
5.3	5	228	229	0.792	0.583	large	Tabu
5.4	5	242	245	0.870	0.740	large	Tabu
5.5	5	211	212	0.885	0.771	large	Tabu
5.6	5	213	214	0.763	0.527	large	Tabu
5.7	5	293	295	0.940	0.880	large	Tabu
5.8	5	288	290	0.790	0.581	large	Tabu
5.9	5	279	280	0.894	0.788	large	Tabu
5.10	5	265	266	0.763	0.527	large	Tabu
6.1	6	138	141	0.821	0.642	large	Tabu
6.2	6	146	148	0.925	0.849	large	Tabu
6.3	6	145	148	0.772	0.544	large	Tabu
6.4	6	131	131	0.742	0.484	large	Tabu
6.5	6	161	164	0.769	0.538	large	Tabu
A.1	A	254	258	0.938	0.876	large	Tabu
A.2	A	252	256	0.868	0.736	large	Tabu
A.3	A	233	237	0.932	0.863	large	Tabu
A.4	A	234	239	0.952	0.905	large	Tabu
A.5	A	236	239	0.954	0.908	large	Tabu
B.1	B	69	70	0.763	0.526	large	Tabu
B.2	B	76	76	0.710	0.419	medium	Tabu
B.3	B	80	81	0.919	0.839	large	Tabu
B.4	B	79	80	0.796	0.591	large	Tabu
B.5	B	72	72	0.667	0.333	medium	Tabu
C.1	C	227	232	0.908	0.815	large	Tabu
C.2	C	220	224	0.914	0.828	large	Tabu
C.3	C	244	249	0.922	0.844	large	Tabu
C.4	C	219	224	0.879	0.758	large	Tabu
C.5	C	215	218	0.890	0.780	large	Tabu
D.1	D	60	62	0.819	0.638	large	Tabu
D.2	D	67	67	0.719	0.437	medium	Tabu
D.3	D	73	74	0.814	0.627	large	Tabu
D.4	D	62	62	0.667	0.333	medium	Tabu
D.5	D	61	61	0.665	0.331	medium	Tabu
NRE.1	NRE	29	29	0.613	0.226	small	Tabu
NRE.2	NRE	30	31	0.847	0.694	large	Tabu
NRE.3	NRE	27	28	0.854	0.708	large	Tabu
NRE.4	NRE	28	29	0.807	0.614	large	Tabu
NRE.5	NRE	28	28	0.586	0.172	small	Tabu
NRF.1	NRF	14	14	0.661	0.323	small	Tabu
NRF.2	NRF	15	15	0.586	0.172	small	Tabu
NRF.3	NRF	14	15	0.777	0.554	large	Tabu
NRF.4	NRF	14	15	0.763	0.527	large	Tabu
NRF.5	NRF	14	14	0.636	0.272	small	Tabu
NRG.1	NRG	176	181	0.856	0.713	large	Tabu
NRG.2	NRG	156	160	0.901	0.802	large	Tabu
NRG.3	NRG	168	174	0.943	0.887	large	Tabu
NRG.4	NRG	173	177	0.852	0.704	large	Tabu
NRG.5	NRG	170	174	0.906	0.813	large	Tabu
NRH.1	NRH	65	67	0.862	0.724	large	Tabu
NRH.2	NRH	64	66	0.849	0.699	large	Tabu
NRH.3	NRH	61	62	0.843	0.686	large	Tabu
NRH.4	NRH	59	61	0.882	0.763	large	Tabu
NRH.5	NRH	55	58	0.848	0.696	large	Tabu

**Table 23:** Comparative performance of BHGS and the compared metaheuristics.

Algorithm	Avg. RPD (%) ^a^	Optimal Instances (%) ^b^	Avg. Rank ^c^
BHGS	0.009	97.8	1.89
BDOA	0.023	93.3	1.93
PSA	19.100	15.6	4.64
SCA	19.437	15.6	4.76
GWO	19.365	15.6	4.79
BAOA	2.756	20.0	4.90
BGO	20.089	15.6	5.09

^a^ Average RPD across the 45 benchmark instances included in the comparative analysis, indicating how far, in percentage terms, the algorithm’s solution falls from the known optimum, on average. Closer to 0 is better; ^b^ percentage of the 45 benchmark instances included in the comparative analysis in which the algorithm reached the known optimum exactly (RPD = 0); ^c^ average ranking of the algorithm across the same 45 benchmark instances. For each instance, the seven algorithms are ordered from best to worst.

**Table 24:** Friedman test results across the seven compared algorithms.

Statistic	Value
$\chi^{2}$ (Friedman) ^a^	169.114
Degrees of freedom ^b^	6
*p*-value ^c^	$6.93\times 10^{-34}$
Decision ^d^	Reject $H_{0}$ (significant global differences)

^a^ Test statistic based on rank differences across algorithms; higher values indicate stronger evidence of unequal performance; ^b^ degrees of freedom for the test, computed as (number of algorithms − 1) = 6; ^c^ probability of observing differences this large under equal performance ($\approx 10^{-34}$: very strong evidence against $H_{0}$); ^d^ whether $H_{0}$ (equal performance) is rejected at α=0.05.

**Table 25:** Wilcoxon signed-rank post hoc test, BHGS versus remaining algorithms (Holm corrected).

Comparison ^a^	Raw *p*-Value ^b^	Holm-Corrected *p*-Value ^c^	Significant (α = 0.05) ^d^
BHGS vs. BDOA	0.180	0.180	No
BHGS vs. BAOA	$1.68\times 10^{-7}$	$4.64\times 10^{-7}$	Yes
BHGS vs. SCA	$7.74\times 10^{-8}$	$4.64\times 10^{-7}$	Yes
BHGS vs. PSA	$7.74\times 10^{-8}$	$4.64\times 10^{-7}$	Yes
BHGS vs. GWO	$7.74\times 10^{-8}$	$4.64\times 10^{-7}$	Yes
BHGS vs. BGO	$7.74\times 10^{-8}$	$4.64\times 10^{-7}$	Yes

^a^ The pair being compared—always BHGS (the proposed method) against one of the other five algorithms; ^b^ The Wilcoxon signed-rank test’s *p*-value for that pair, unadjusted. With six simultaneous comparisons, using this value alone would inflate the risk of false positives; ^c^ The *p*-value adjusted using the Holm [35] correction, which controls that risk when comparing multiple pairs at once. This is the value used to decide significance; ^d^ Whether the Holm-corrected *p*-value falls below 0.05 (“Yes”) or not (“No”).

## Data Availability

The original contributions presented in the study are included in the article; further inquiries can be directed to the corresponding authors.

## Abbreviations

The following abbreviations are used in this manuscript:

HGS	Hunger Games Search
BHGS	Binary Hunger Games Search
ARSM	The Adaptive Repair Selection Mechanism
TS	Tabu Search
CI	Convergence Iteration
MAB	Multi-Armed Bandit
SCP	Set Covering Problem
DS	Discretization Scheme
ORE	Repair Operators
RPD	Relative Percentage Deviation
CV	Coefficient of Variation
V3-Elit	V-shaped elitist binarization
